# Weight stigma and engagement in physical health behaviours among young adults in India

**DOI:** 10.1080/21642850.2026.2687229

**Published:** 2026-06-10

**Authors:** Ankita Sehrawat, Susan M. Sherman, Nicola J. Buckland, Chantelle Wood

**Affiliations:** a School of Psychology, University of Sheffield, Western Bank, Sheffield, United Kingdom

**Keywords:** Weight stigma, health behaviour, India, stress, self-esteem

## Abstract

**Background:**

Weight stigma - bias, prejudice, and discrimination based on weight - affects engagement in physical health behaviours in Western populations. Effects on individuals from South Asia, particularly India, are less well understood.

**Objective:**

This study examined whether weight stigma is associated with young Indian adults’ engagement in physical health behaviours and explored potential moderators and mediators.

**Methods:**

781 adults (18–26 years) completed an online survey. Multiple regression analyses assessed whether experienced (EWS), anticipated (AWS), and internalised (IWS) weight stigma is associated with engagement in physical health behaviours while controlling for self-reported BMI, gender, age, and income, whether effects were mediated by stress and self-esteem, and moderated by perceived weight status and BMI.

**Results:**

EWS, AWS and IWS were significantly associated with greater engagement in unhealthy and extremely unhealthy weight control behaviours, alcohol consumption, and binge eating with and without loss of control. EWS was also associated with greater cigarette use. AWS and EWS were associated with lower physical activity. While stress more consistently mediated the relationship between weight stigma and health behaviour, self-esteem emerged as a novel mediator. Perceived weight status was a more consistent moderator than BMI.

**Discussion:**

This is the first study to provide evidence that weight stigma is associated with greater engagement in unhealthy behaviours and less engagement in healthy behaviours in young Indian adults. Mediation by stress supports COBWEBS and the Weight-Based Social Identity Threat Model, while self-esteem emerged as a novel mediator. These findings offer insights into mechanisms linking weight stigma to physical health behaviours and the impacts of weight stigma within a non-Western setting.

## Introduction

Weight stigma is defined as negative attitudes, beliefs and behaviours that devalue people based on their weight (Brewis et al., 2018; Rubino et al., 2020). The prevalence and impact of weight stigma have been widely studied in Western societies. Reviews indicate that weight stigma has negative impacts on mental and physical health (Emmer et al., 2020; Romano et al., 2023; Zhu et al., 2022). Contrary to the popular belief that weight stigma motivates individuals to engage in healthier behaviours (Callahan, 2013), weight stigma is associated with greater engagement in unhealthy behaviours such as disordered eating (Brown et al., 2016), substance use (Puhl et al., 2019; Simone et al., 2019; Sutin & Terracciano, 2017) and unhealthy weight-control strategies (e.g. using laxative, diet pills) (Gerend et al., 2023; Simone et al., 2019), and less engagement in healthy behaviours such as physical activity (Pearl et al., 2021; Pont et al., 2017) and engagement with healthcare (Byrd et al., 2023; Wetzel & Himmelstein, 2023). Theoretical frameworks implicate the role of stress in the relationship between weight stigma and health behaviours. Tomiyama’s Cyclic Obesity/Weight-Based Stigma (COBWEBS) model (e.g. Tomiyama, 2014) depicts a vicious cycle where experiences of weight stigma function as a chronic stressor that increases cortisol levels, triggering comfort eating and subsequent weight gain, which in turn reinforces the cycle of stigma. The Weight-Based Social Identity Threat Model (e.g. Hunger et al., 2015) proposes that belonging to a higher-weight social group creates fear and anticipation of future stigma, which triggers psychological distress and elevated cortisol, leading to unhealthy food consumption. The model also argues that coping with weight stigma-induced stress taxes executive functioning and depletes the mental resources necessary for self-regulation of other health behaviours such as physical activity. While the model explicitly focuses on eating behaviour and physical activity, the underlying mechanisms should generalise to any behaviour that relies on self-regulation. Both models are supported by empirical research, showing that stress mediates the relationship between weight stigma and disordered eating (e.g. Huang et al., 2024), physical activity (Hackett et al., 2023) and avoidance of healthcare (Mensinger et al., 2018).

Self-esteem - an individual's evaluative judgement of their worth (Rosenberg, 1965) - is one of the most consistent psychological consequences of weight stigma, mediating effects on mental health (Sikorski et al., 2015). However, no studies have examined whether self-esteem mediates the relationship between weight stigma and engagement in physical health behaviour. Given the effects of weight stigma on self-esteem, alongside evidence that higher self-esteem is associated with healthier behaviours (Arsandaux et al., 2020), self-esteem seems a plausible mediator.

Critically, most of the weight stigma literature is Western-centric, resulting in widespread calls for research to explore the prevalence of weight stigma and its impact in non-Western countries (e.g. Eggerichs et al., 2024; Zhao et al., 2024; Zhu et al., 2022). Evidence suggests that anti-fat attitudes are emerging worldwide, even in cultures that have traditionally embraced fat-positivity (e.g. Brewis et al., 2018; Jiwanmall et al., 2022). A recent scoping review on weight stigma in non-Western societies indicated that while experiences of stigma were varied, some countries, including India, show signs of emerging weight stigma (Eggerichs et al., 2024). Critically, while the review identified four studies involving Indian populations, these primarily focused on body image or bullying of individuals with obesity and did not quantitatively measure weight stigma or its relationship with engagement in physical health behaviour.

India has the largest population in the world, with 1.46 billion inhabitants (Worldometer, 2025). India has a high prevalence of overweight and obesity, with 70% of India's urban population classified as living with overweight or obesity (The Economic Times, 2024). While historically, curvier bodies were admired in India (Gelles, 2011), colonisation and increased globalisation have significantly changed beauty standards. Post-colonisation, British ideals of beauty that emphasise thinness have gained prominence (Wardhani et al., 2017), further reinforced by Indian media that promote Western beauty ideals (Nagar & Virk, 2017). For example, 86% of college-going women in India preferred slimness (Latha et al., 2006). Given this context, it is important to investigate the association between weight stigma and engagement in physical health behaviour in India.

### The current study

The current research investigated the relationship between weight stigma and engagement in physical health behaviours in young Indian adults. Gerend et al. (2023) emphasise the importance of exploring how anticipated and internalised weight stigma influence health behaviours, even in the absence of actual experienced stigma. Therefore, this study examined associations between engagement in physical health behaviour and experienced (direct instances of being treated unfairly or discriminated against), anticipated (fear of being rejected or treated unfairly), and internalised (self-devaluation or applying negative stereotypes to oneself) weight stigma, as defined by Major et al. (2020). Previous research on weight stigma and physical health behaviour has primarily focused on eating behaviours and physical activity (e.g. Puhl & Suh, 2015; Zhu et al., 2022). However, evidence shows that weight stigma may influence a broader range of physical health behaviours, including alcohol use (e.g. Wu & Berry, 2018), smoking (Sutin & Terracciano, 2017), and maladaptive weight control strategies (Simone et al., 2019). We therefore included a broader range of physical health behaviours.

In line with the COBWEBS (e.g. Tomiyama, 2014) and Weight-Based Social Identity Threat (e.g. Hunger et al., 2015) models, the current study also evaluated whether stress mediates the relationship between weight stigma and physical health behaviour. Given the separate bodies of literature demonstrating a relationship between weight stigma and self-esteem (e.g. see Sikorski et al., 2015) and self-esteem and health behaviours (e.g. Arsandaux et al., 2020), we also examined for the first time whether self-esteem mediates the relationship between weight stigma and physical health behaviours. Understanding these pathways can help pinpoint modifiable targets for intervention that may buffer the negative impact of weight stigma on health.

Finally, given evidence that impacts of weight stigma are stronger for individuals with higher BMI as well as those who perceive themselves to be overweight (e.g. Hunger et al., 2018; Jackson et al., 2016), we examined whether these moderator effects replicate in the Indian context.

The current study focuses on young adults (aged 18–26), as a particularly important demographic. India has one of the youngest populations globally, with 29.3% of its population aged 15–29 years - an estimated 345 million people by 2036 (Ministry of Statistics and Programme Implementation, 2022). Young Indians are increasingly influenced by Western beauty ideals that promote thinness, especially through media exposure (Singh & Gadiraju, 2020). As such, weight stigma may disproportionately affect this population.

Based on the literature discussed, the current study hypothesised that higher experienced, anticipated and internalised weight stigma would be associated with greater engagement in unhealthy behaviours and lower engagement in healthy behaviours; that this association would be mediated by stress and self-esteem; and moderated by BMI and perceived weight status.

## Method

The study was pre-registered on OSF [https://osf.io/3atdb/overview?view_only=6ee049f6f8774d45985c36d441e2ad5c], and all materials and data are openly available on the OSF [https://osf.io/dax7b/overview].

### Design

The current study used a cross-sectional survey research design. Dependent variables were healthy/unhealthy weight control strategies, physical activity, binge eating with and without loss of control, and substance use. Stress and self-esteem were examined as mediators, and perceived weight status and BMI as moderators.

### Participants

A power analysis (G*Power 3.1: Faul et al., 2009) assuming a small effect size (f2 = .02), ɑ = .05, and power = .80, indicated that the required sample size was 653. A total of 781 young adults from India (*M* age = 19.93 years, *SD* = 1.72) participated in the study. The majority were female (79.6%), and average body mass index (BMI) was 22.72 kg/m² (*SD =* 4.75). Participants were recruited via social media and collaboration with university academics in India, who distributed the survey within their networks. We also invited participants to share the recruitment invitation with their networks. Eligibility criteria included: a) aged between 18-26 years, b) proficient in English, c) access to a smartphone or laptop with internet connectivity. No remuneration was provided for participation.

### Procedure

Participants provided informed consent before proceeding with the study. The survey was administered via Qualtrics (Provo, UT). The order of questions was as follows: demographic information, anthropometric data, health behaviours, stress, self-esteem, and weight stigma. The study was approved by the University of Sheffield Ethics Committee.

### Measures

Measures of physical health behaviour, stress, and self-esteem were drawn from the Project EAT 2018 Survey (Eisenberg et al., 2019; Larson et al., 2013), which itself adapted items from previously validated instruments. Some response options were adapted to ensure cultural relevance (e.g. currency, metrics for weight measurement). A full list of included measures is available on OSF [https://osf.io/dax7b/overview].

### Experience of weight stigma (EWS)

EWS was assessed using three items developed by Puhl et al. (2011). Participants indicated whether they had experienced any of the following due to their weight: a) being teased, b) being treated unfairly, c) being discriminated against, with a yes/no response. Following Himmelstein et al. (2017), responses were combined to create a binary variable, such that participants who responded ‘yes’ to at least one item were coded as 1 (EWS), whereas those who responded ‘no’ to all items were coded as 0 (no EWS).

### Anticipated weight stigma (AWS)

AWS was assessed using five items developed by Hunger and Major (2015). Participants rated their agreement with the statements (e.g. *“I am afraid that other people will reject me because of my weight”*) on a 7-point scale (1 [strongly disagree] to 7 [strongly agree]). The sum of all five items was calculated, with a higher score reflecting greater AWS. Cronbach’s alpha indicated satisfactory internal consistency (*α* = .96).

### Internalised weight stigma (IWS)

IWS was assessed using the 11-item Modified Weight Bias Internalisation Scale (WBIS-M), a version of the Weight Bias Internalisation Scale (Durso & Latner, 2008) modified by Pearl and Puhl (2014) to cover all weight statuses. Participants rated their agreement with the statements (e.g. *"My weight is a major way that I judge my value as a person")* on a 7-point scale (1 [strongly disagree] to 7 [strongly agree]). Responses were averaged, with a higher value indicating greater IWS. Cronbach’s alpha indicated satisfactory internal consistency (*α* = .93).

### Stress

Stress was assessed using the 4-item version of the Perceived Stress Scale (PSS, Cohen & Williamson, 1988), which measures the extent to which participants have experienced stress in the last month (e.g. “*felt that things were going your way?”*). Responses are made on a 4-point scale (0 [never] to 4 [very often]). Two items were reverse scored. The total score was then calculated by summing responses across the four items. Higher scores indicated higher stress. Cronbach’s alpha indicated that internal consistency was just under a satisfactory level (*α* = .66).

### Self esteem

Self-esteem was assessed using the 10-item Rosenberg Self-Esteem Scale (Rosenberg, 1965). Participants rated their agreement with the statements (e.g. “*On the whole, I am satisfied with myself*”) on a 4-point scale ranging (1 [strongly disagree] to 4 [strongly agree]). Five items were reverse scored. The total score was then calculated by summing responses across all the items, with higher scores indicating higher self-esteem (*α* = .84).

### Physical activity (PA)

PA was assessed using the Godin Leisure-Time Exercise Questionnaire (LTEQ; Godin & Shephard, 1985). Participants reported the number of times per week they performed physical activities lasting more than 15 minutes in three categories: strenuous PA (e.g. running, basketball), which involves rapid heart rate and significant effort, moderate PA (e.g. fast walking, skiing), which is less intense but still requires effort, and mild PA (e.g. easy walking, golf), which involves minimal exertion. A moderate-to-strenuous PA score (MSPA) was calculated following the formula provided by Godin and Shephard (1985), where a higher score indicates greater physical activity.

### Healthy and unhealthy weight control behaviour

Healthy and unhealthy weight control behaviours were assessed by asking participants whether they had engaged *“in any of the following activities to lose weight or prevent weight gain in the past year*”, with a yes/no response. The question was adapted from Neumark-Sztainer et al. (2002) who modified the Pound of Prevention Survey (Jeffery & French, 1999). Healthy weight control behaviours (HWCB) included: *"Exercised," "Ate more fruits and vegetables," "Ate less high-fat foods," "Ate fewer sweets," "Drank less soda," "Drank more water,"* and *"Watched my portion sizes" (* *α* = .84*).* Unhealthy weight control behaviours (UWCB) included: *"Fasted," "Ate very little food," "Used a food substitute," "Skipped meals," "Smoked more cigarettes," "Took diet pills," "Made myself vomit," "Used laxatives," and "Used diuretics" (* *α* = .84*).*

Additionally, following Neumark-Sztainer et al. (2002), specific items from the UWCB measure were categorised as extremely unhealthy weight control behaviours (EUWCB), namely: *"Took diet pills," "Made myself vomit," "Used laxatives," and "Used diuretics".* Healthy, unhealthy, and extremely unhealthy weight control behaviour scores were calculated using the sum of ‘yes’ responses, where a higher score indicated engagement in a greater number of these behaviours.

### Binge eating with and without loss of control

Binge eating behaviours were assessed using two items from the Questionnaire on Eating and Weight Patterns-Revised (Yanovski, 2014). Participants first responded to the statement *“In the past year, have you ever eaten so much food in a short period of time that you would be embarrassed if others saw you?”* with a yes/no response.

Those who responded ‘yes’ were further asked to respond to the statement *“During the times when you ate this way, did you feel you couldn’t stop eating or control what or how much you were eating?”,* with a yes/no response. Participants who answered ‘yes’ to both questions were classified as engaging in binge eating with loss of control, whereas those who responded ‘yes’ to the first question only were classified as engaging in binge eating without loss of control.

### Substance use

Substance use was assessed using items adapted by Neumark-Sztainer et al. (2002) from the Voice of Connecticut Youth Survey (Sherwood et al., 2002). Participants indicated whether they had used any of the following substances in the past 12 months: cigarettes, beer, wine, hard liquor, marijuana, or other drugs (e.g. cocaine, heroin, etc.). Response options were *"never"*, *"a few times", "monthly", "weekly",* and *"daily".* The original survey did not cover e-cigarette use. Due to the growing popularity of e-cigarettes in India despite regulatory bans (Pettigrew et al., 2023), this item was added.

### Demographics and anthropometric data

Participants self-reported their age, gender, educational level, income level, height (in feet/inches), and weight (in kilograms). Body Mass Index (BMI) was calculated using the Centres for Disease Control and Prevention (Kuczmarski et al., 2002) guidelines.

### Weight perception

To assess subjective weight status, participants rated their current weight status *“very underweight”, “underweight”, “just about right”, “overweight”, and “very overweight.”* (Hunger et al., 2020; Lee et al., 2021).

### Statistical analysis

All analyses were conducted using *IBM SPSS Statistics for Windows* (Version 29.0) (IBM Corp, 2021). Descriptive statistics were computed to summarise sample characteristics, along with correlations between variables. Regression models were conducted to investigate the associations between weight stigma and each health behaviour, using linear regression for continuous outcomes (e.g. physical activity) and logistic regression for binary outcomes (e.g. binge eating). All models used the Enter method. The type of weight stigma was entered as the predictor and the health behaviour as the outcome. Age, gender, BMI, and income levels were included as covariates to test the unique association between weight stigma and health behaviours.

All variables were assessed for normality using histograms, Q-Q plots, and skewness and kurtosis statistics. Variables with skewed distributions (EUWCB, marijuana use, and drug use) were transformed using the natural log. However, marijuana and drug use remained skewed after transformation and were therefore excluded from regression analyses; only descriptive statistics are presented for these variables.

For health behaviours that were significantly associated with weight stigma in the regression analyses, mediation and moderation analyses were conducted using the PROCESS macro for SPSS (Hayes, 2017). Mediation was tested using Model 4 and moderation using Model 1. Bootstrapping procedures with 5,000 resamples were employed to estimate 95% bias-corrected and accelerated (BCa) confidence intervals for indirect effects. Mediation and moderation effects were considered statistically significant if the confidence interval did not include zero. All mediation and moderation models controlled for age, gender, BMI, and income. Unadjusted regression analyses are available in the supplementary material.

### Ethics statement

The Ethics approval was obtained from the ethics committee of the School of Psychology, University of Sheffield [065265].

## Results

47.1% (*n* = 368) of participants reported experiencing teasing, unfair treatment or discrimination at least once in their lifetime The average score on the WBIS-M was 2.74 (1.83), and AWS was 11.37 (3.82), indicative of low-to-moderate levels of internalised and anticipated weight stigma in the sample. See Supplementary Materials Tables 1 and 2 for full descriptive statistics and correlations between variables.

### Association between EWS and health behaviours

Positive associations were observed between EWS and binge eating with loss of control (B = 0.62, OR = 1.85, 95% CI [1.27, 2.72], *p* = .001) and binge eating without loss of control (B = 0.58, OR = 1.78, 95% CI [1.28, 2.48], *p* = .001), EUWCB (*β* = 0.18, *p* = .001), UWCB (*β* = 0.15, *p* = .001), alcohol consumption (*β* = 0.12, *p* < .001), and cigarette use (*β* = 0.09, *p* = .014). A small negative association was found between EWS and PA (*β* = -0.12, *p* = .002). No significant associations were observed between EWS and e-cigarette use or HWCB.

### Association between AWS and health behaviours

Positive associations were observed between AWS and UWCB (*β* = 0.29, *p* = .001) alcohol consumption (*β* = 0.20, *p* = .001), EUWCB (*β* = 0.18, *p* < .001), binge eating with loss of control (B = 0.06, OR = 1.06, 95% CI [1.04, 1.09], *p* = .001), and binge eating without loss of control (B = 0.06, OR = 1.06, 95% CI [1.03, 1.08], *p* = .001). A small negative association was found between AWS and PA (*β* = -0.14, *p* = .001). No significant associations were observed between AWS and HWCB, cigarette use, or e-cigarette use.

### Association between internalised weight stigma and health behaviours

IWS was positively associated with UWCB (*β* = 0.27, *p =* .001), EUWCB (*β* = 0.26, *p =* .001) alcohol consumption (*β* = 0.14, *p =* .001), binge eating with loss of control (B = 0.03, OR = 1.03, 95% CI [1.02, 1.04], *p =* .001), and binge eating without loss of control (B = 0.02, OR = 1.02, 95% CI [1.01, 1.03], *p =* .001). No significant associations were observed between IWS and HWCB, PA, cigarette use, or e-cigarette use.

### Mediation

#### Experienced weight stigma

**Figure 1.**
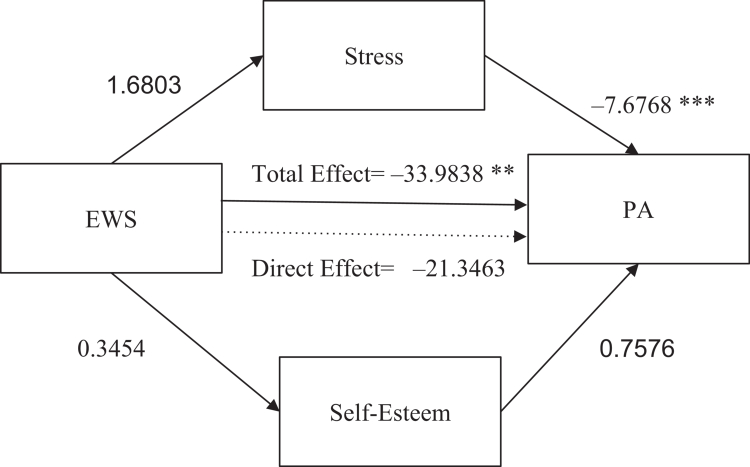
Mediation model showing the effect of EWS on PA via self-esteem and stress.

For the relationship between EWS and PA (Figure 1), results were consistent with full mediation by stress, such that the direct effects of EWS became non-significant after accounting for stress.

**Figure 2.**
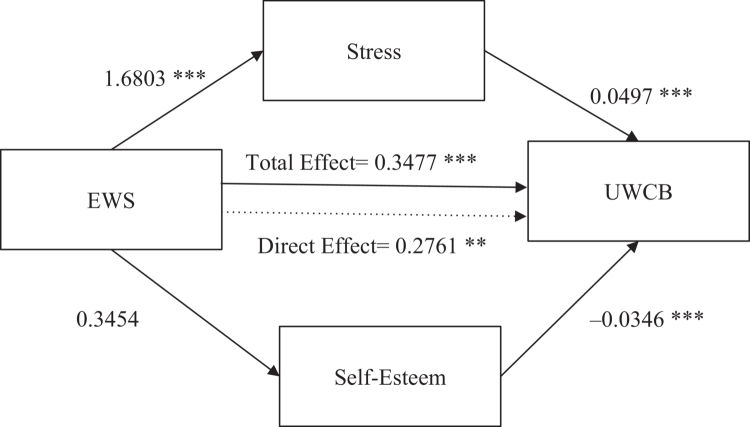
Mediation model showing the effect of EWS on UWCB via self-esteem and stress.

**Figure 3.**
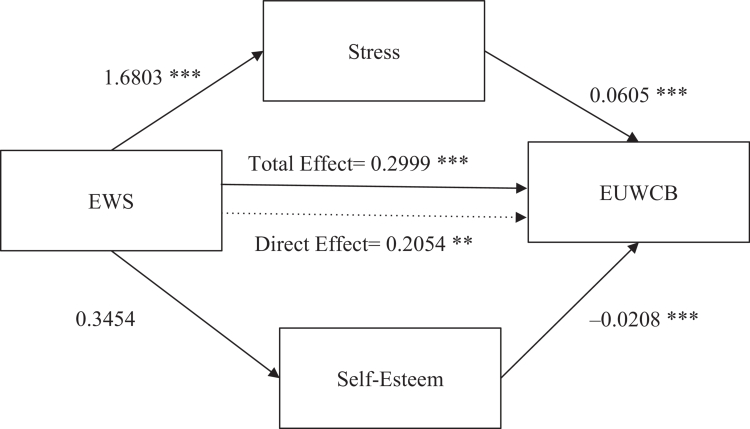
Mediation model showing the effect of EWS on EUWCB via self-esteem and stress.

**Figure 4.**
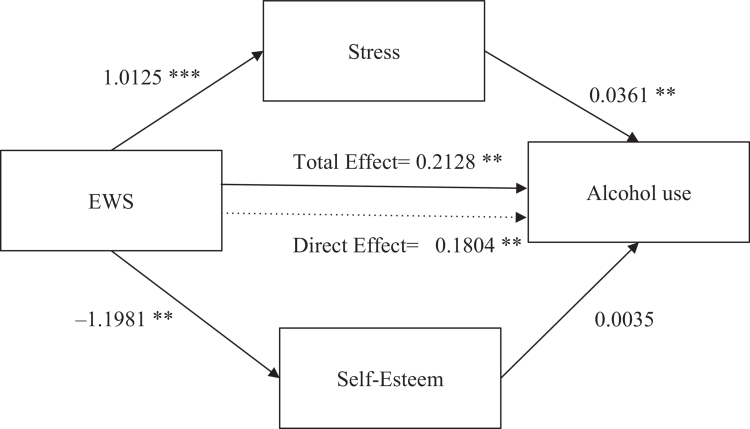
Mediation model showing the effect of EWS on alcohol use via self-esteem and stress.

**Figure 5.**
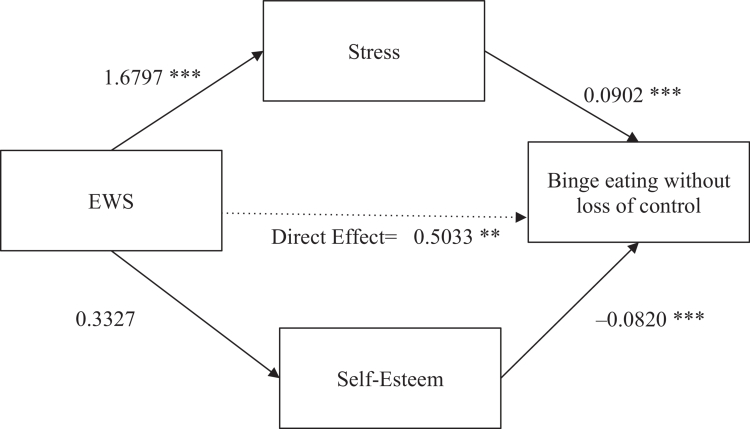
Mediation model showing the effect of EWS on binge eating without loss of control via self-esteem and stress.

**Figure 6.**
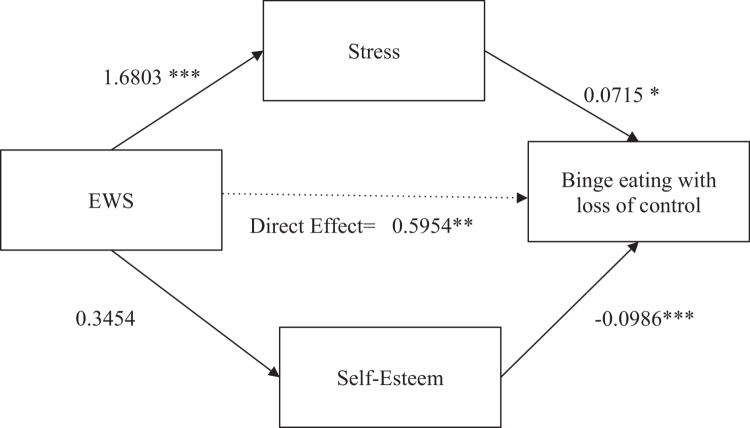
Mediation model showing the effect of EWS on binge eating with loss of control via self-esteem and stress.

Partial mediation by stress was observed in the relationships between EWS and UWCB (Figure 2), EUWCB (Figure 3) and alcohol use (Figure 4), such that EWS was associated with higher stress and higher stress was associated with greater engagement in these behaviours. For binge eating without loss of control (Figure 5), partial mediation was observed, with EWS associated with increased stress and decreased self-esteem, both of which partially mediated the outcome. However, for binge eating with loss of control (Figure 6), only decreased self-esteem partially mediated the relationship.

No significant mediation by either stress or self-esteem was found for the relationship between EWS and cigarette use.

#### Anticipated weight stigma

**Figure 7.**
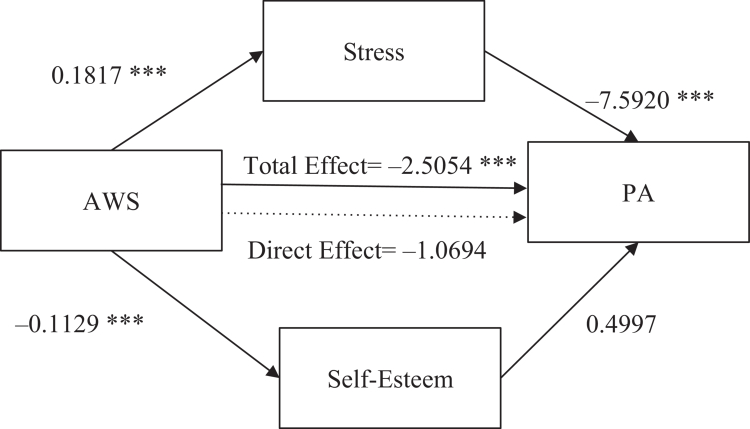
Mediation model showing the effect of AWS on PA via self-esteem and stress.

Full mediation by stress was observed in the relationships between AWS and PA (Figure 7), such that the direct effects of AWS became non-significant after accounting for stress.

**Figure 8.**
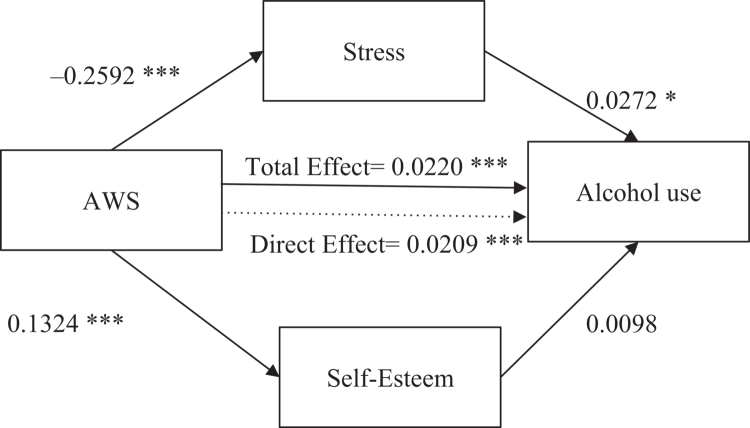
Mediation model showing the effect of AWS on alcohol use via self-esteem and stress.

**Figure 9.**
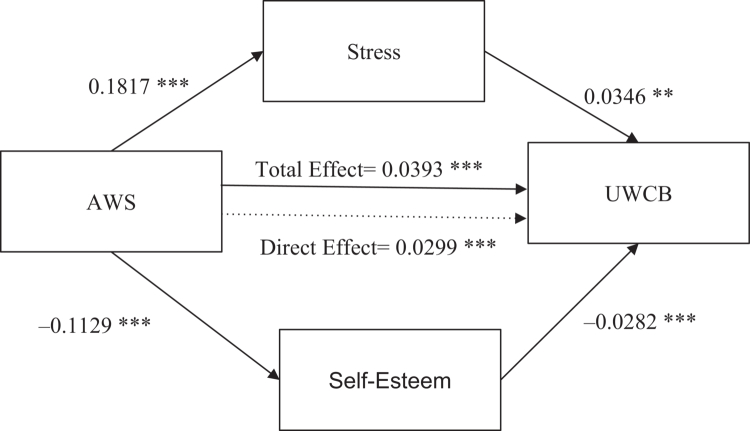
Mediation model showing the effect of AWS on UWCB via self-esteem and stress.

**Figure 10.**
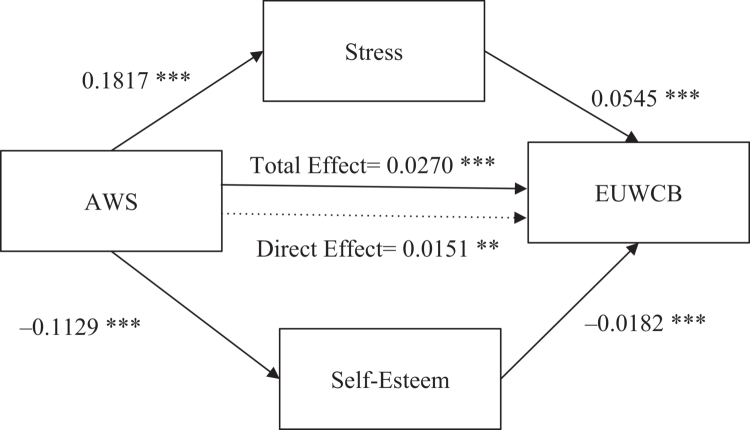
Mediation model showing the effect of AWS on EUWCB via self-esteem and stress.

**Figure 11.**
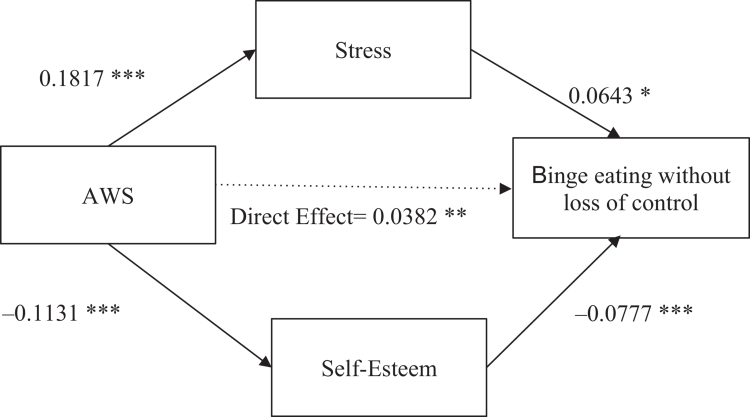
Mediation model showing the effect of AWS on binge eating without loss of control via self-esteem and stress.

**Figure 12.**
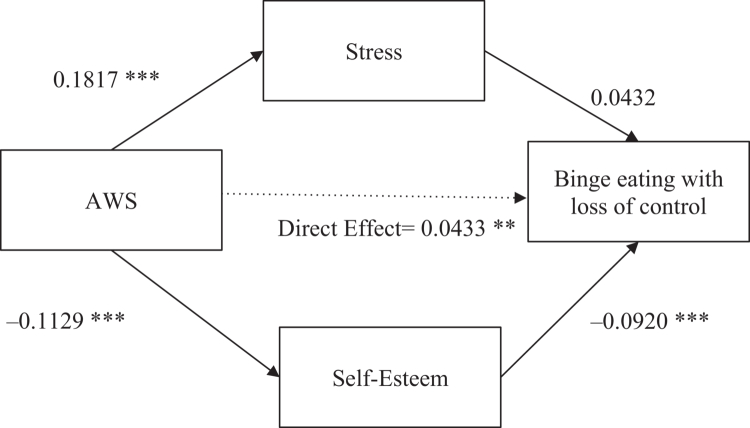
Mediation model showing the effect of AWS on binge eating with loss of control via self-esteem and stress.

Partial mediation by stress was observed for AWS and alcohol use (Figure 8), with increased stress associated with increased alcohol use. For UWCB (Figure 9), EUWCB (Figure 10), and binge eating without loss of control (Figure 11), higher AWS was associated with increased stress and decreased self-esteem, both partially mediating the outcomes. For binge eating with loss of control (Figure 12), only decreased self-esteem partially mediated the relationship.

#### Internalised weight stigma

**Figure 13.**
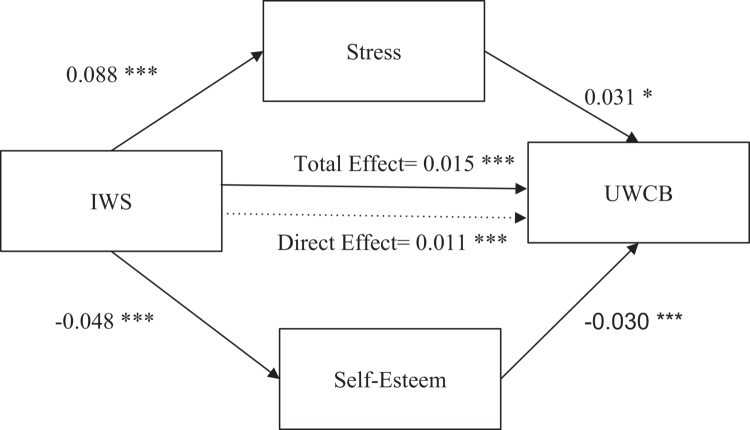
Mediation model showing the effect of IWS on UWCB via self-esteem and stress.

**Figure 14.**
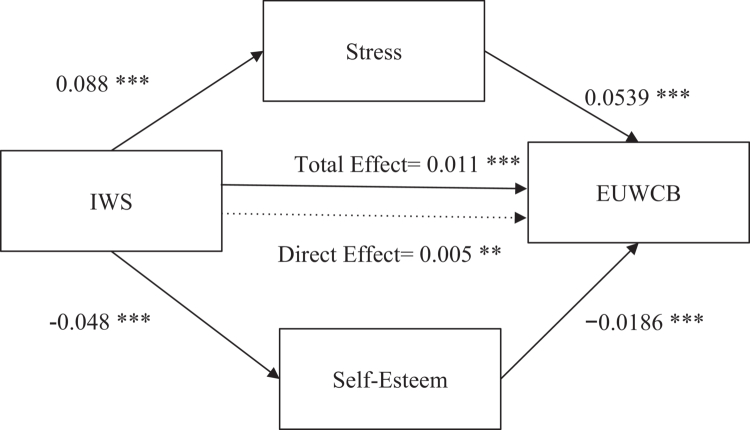
Mediation model showing the effect of IWS on EUWCB via self-esteem and stress.

**Figure 15.**
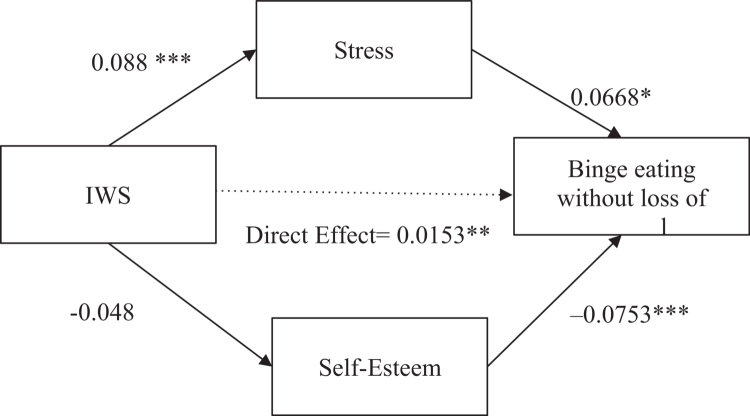
Mediation model showing the effect of IWS on binge eating without loss of control via self-esteem and stress.

Partial mediation was observed in the relationships between IWS and UWCB (Figure 13), EUWCB (Figure 14), and binge eating without loss of control (Figure 15), such that both the direct effects of IWS and indirect effects via self-esteem and stress were significant.

**Figure 16.**
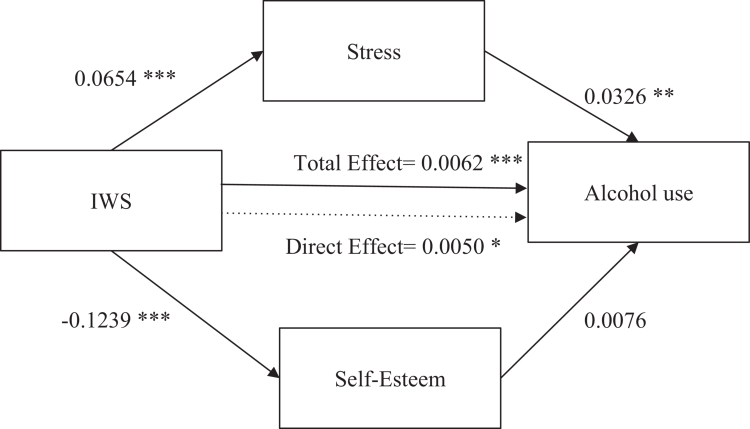
Mediation model showing the effect of IWS on alcohol use via self-esteem and stress.

**Figure 17.**
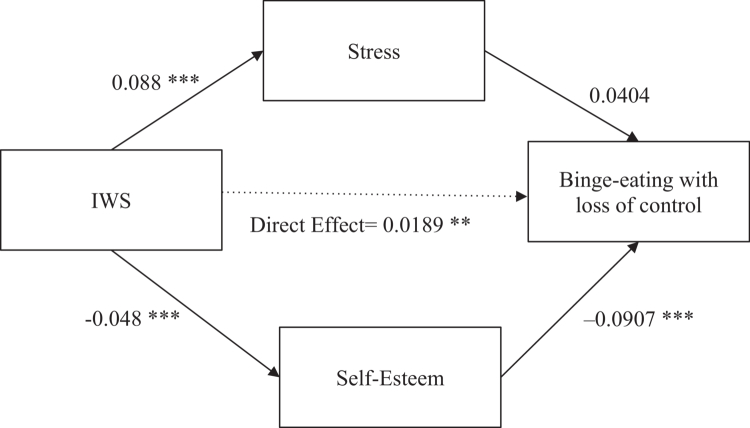
Mediation model showing the effect of IWS on binge eating with loss of control via self-esteem and stress.

Stress was a significant mediator in the relationship between IWS and alcohol use (Figure 16), indicating that IWS was associated with higher alcohol consumption through increased stress, with no significant mediation via self-esteem. While self-esteem was a significant mediator in the relationship between IWS and binge eating with loss of control (Figure 17), reduced self-esteem explained the indirect effect of IWS on binge eating with loss of control.

### Moderation

For parsimony, only significant moderators are reported below. See Supplementary Materials for non-significant results.

### Perceived weight status

Perceived weight status significantly moderated the association between EWS, AWS and IWS, with UWCB and EUWCB, such that the positive associations between EWS and both UWCB (*b* = 0.18, *p* = .03) and EUWCB (*b* = 0.22, *p =* .001); AWS and both UWCB (*b* = 0.01, *p* = .006) and EUWCB (*b* = 0.02, *p =* .001); and IWS and both UWCB (*b* = 0.01, *p* = .008) and EUWCB (*b* = 0.01, *p =* .001) were stronger among individuals who perceived themselves as overweight.

Perceived weight status significantly moderated the association between EWS and AWS with binge eating without loss of control, such that the positive associations between EWS and binge eating without loss of control (*b* = 0.66, *p =* .001) and AWS and binge eating without loss of control (*b* = 0.02, *p* = .032) were stronger among individuals who perceived themselves as overweight. There was no significant moderation of the association between IWS and binge eating without loss of control.

All other moderation effects were specific to one weight stigma variable. Perceived weight status significantly moderated the association between EWS and binge eating with loss of control (*b* = 0.73, *p* = .002), such that the positive association was stronger among individuals who perceived themselves as overweight; and EWS and cigarette use (*b* = –0.21, *p* = .017), such that the positive association was stronger among individuals who perceived themselves as lower weight. There was no significant moderation of the association between AWS and IWS and these health behaviours. Perceived weight status also significantly moderated the association between IWS and PA (*b* = –0.65, *p* = .020), such that the positive association was weaker among individuals who perceived themselves as overweight. There was no significant moderation of the association between EWS and AWS and these health behaviours.

Perceived weight status did not moderate any relationship between weight stigma variables and HWCB, alcohol consumption, e-cigarettes, and binge eating with loss of control.

### BMI

BMI significantly moderated the association between EWS, AWS and IWS with EUWCB such that the positive associations between EWS and EUWCB (*b* = 0.04, *p* = .005), AWS and EUWCB (*b* = 0.003, *p =* .001) and IWS and EUWCB (*b* = 0.002, *p =* .001) were stronger among individuals with higher BMI.

BMI significantly moderated the associations between EWS and binge eating without loss of control (*b* = 0.08, *p* = .045) and binge eating with loss of control (*b* = 0.09, *p* = .038), such that positive associations were stronger among individuals with higher BMI. BMI also significantly moderated the association between IWS and UWCB (*b* = 0.001, *p* = .010), such that the positive association was stronger among individuals with higher BMI; and IWS and HWCB (*b* = –0.0045, *p* = .027) and PA (*b* = –0.13, *p* = .024), such that the positive association was stronger among individuals with lower BMI.

## Discussion

This study found that weight stigma was consistently associated with a range of health behaviours after adjusting for BMI, age, gender, and income. That is, greater EWS, AWS, and IWS were significantly associated with greater engagement in UWCB, EUWCB, binge eating with and without loss of control, and higher alcohol consumption. However, only EWS and AWS were associated with lower engagement in PA, and only EWS was associated with greater cigarette use. None of the weight stigma measures were associated with e-cigarette use and HWCB. These findings mirror patterns reported in Western contexts (Simone et al., 2019; Vartanian & Porter, 2016; Zhao et al., 2024), providing the first evidence that negative associations between weight stigma and engagement in physical health behaviours extend to India.

As predicted, EWS and AWS were associated with lower PA, suggesting that weight stigma may inhibit engagement in health-promoting behaviours. However, IWS showed no such association. This is consistent with Pearl et al. (2021) systematic review, which demonstrated that IWS is more strongly associated with motivational factors such as self-efficacy and enjoyment rather than actual exercise behaviour, but at odds with research demonstrating that individuals with high levels of IWS may engage in exercise as a coping mechanism to manage stress (e.g. Romano et al., 2021).

When considering the relationship between weight stigma and cigarette use, our results are consistent with Himmelstein et al. (2019), who reported an association between smoking and EWS but not IWS. This could be because our sample is predominantly women, who are more likely to use social or emotion-focused coping rather than substance use when managing stress (Graves et al., 2021). Non-significant associations between any form of weight stigma and e-cigarette use are likely due to the low prevalence of e-cigarette use in India, where regulatory bans limit their availability (Pettigrew et al., 2023). The lack of any relationship between weight stigma and our compound healthy weight control strategy outcome is consistent with the existing literature. For example, Levy et al. (2022) reported that weight stigma is linked to greater use of unhealthy practices, such as excessive exercise, rather than healthy weight control strategies.

Finally, it is important to highlight that effect sizes varied across weight stigma types, particularly for binge eating behaviour. Emerging evidence suggests that EWS has a stronger association with binge eating behaviour. For instance, a recent study found that pressures from healthcare professionals to maintain a healthy weight were associated with binge eating symptoms, but only via the pathway of IWS, whereas exposure to general healthy-weight discourse showed no significant effects on binge eating symptoms (D’Amico et al., 2025). These findings suggest that not all forms of weight stigma have the same impact.

### Mediating role of stress and self-esteem

While the cross-sectional design limits definitive conclusions about causation, results from the current study are consistent with the proposal that stress mediates the relationship between weight stigma and engagement in physical health behaviour - stress statistically fully mediated the associations between all three types of weight stigma on PA and partially mediated the association between weight stigma and UWCB, binge eating without loss of control, and alcohol use. These findings provide empirical support for the COBWEB model (Tomiyama, 2014) and the Weight-Based Social Identity Threat Model (e.g. Hunger et al., 2015), which both centre stress as the causal mechanism underlying the impacts of weight stigma on health. Our findings are also consistent with research demonstrating that individuals exposed to weight stigma experience elevated cortisol levels (Rodriguez et al., 2016; Schvey et al., 2014), which in turn trigger unhealthy behaviours such as higher calorie intake (Tomiyama, 2014).

Self-esteem also statistically mediated the relationship between weight stigma and engagement in UWCB, EUWCB, and binge eating with and without loss of control. To our knowledge, this is the first study to provide empirical evidence for the key role of self-esteem, enhancing knowledge of the psychological processes underpinning stigma-related health risks, and suggesting new paths for intervention. While the existing models discussed primarily implicate stress, our findings suggest that self-esteem is an additional mechanism through which weight stigma can influence health. Theoretical frameworks that integrate the role of self-esteem are therefore needed.

### Moderating role of perceived weight status and BMI

Moderation analyses demonstrate perceived weight status more consistently moderated associations between weight stigma and engagement in health behaviours, relative to BMI - though both were important. Individuals who perceived themselves as overweight exhibited stronger associations between all forms of weight stigma and engagement in both UWCB and EUWCB, and between EWS and AWS with binge eating without loss of control. In contrast, while individuals with higher BMI also exhibited stronger associations between all forms of weight stigma and engagement in EUWCB, BMI only moderated the association between IWS and engagement in UWCB, and between EWS and binge eating without loss of control.

For some health behaviours, perceived weight status and BMI had consistent effects that were limited to a specific form of weight stigma. Perceived weight status and BMI moderated the association between weight stigma and binge eating with loss of control for EWS, but not for AWS or WBI; and moderated the association between weight stigma and PA for WBI, but not for EWS or AWS. Although IWS was not directly associated with engagement in PA, a significant interaction with perceived weight status and BMI emerged, such that IWS was associated with lower PA only in those with higher perceived weight status or BMI. Cigarette use results diverge from the patterns seen for other unhealthy behaviours - experienced weight stigma was only associated with greater cigarette use in people with lower perceived weight status, indicating that behavioural responses to stress differ as a function of perceived weight status. This idea is supported by research showing that alcohol consumption tends to decline as BMI increases among women, indicating a shift toward food as a preferred coping method over substances like alcohol or cigarettes (Kleiner et al., 2004). Finally, associations between weight stigma and alcohol use appeared relatively stable regardless of perceived weight status and BMI.

Together, these findings indicate that perceived weight status may serve as a more reliable and consistent moderator than BMI in understanding the relationship between weight stigma and engagement in physical health behaviour. This aligns with previous research suggesting that subjective weight perception can be more influential than objective measures of weight in predicting health behaviours, particularly disordered eating, weight loss attempts, eating and PA (Haynes et al., 2018; Romano et al., 2018). These findings align with the Weight-Based Social Identity Threat Model (Hunger et al., 2015), which argues that individuals experience weight-based social identity threat (and associated vulnerability to adverse health outcomes) when they categorise themselves as higher-weight (Hudson et al., 2025).

### Limitations and future research

Limitations of the current study include the use of a cross-sectional design, which prevents drawing concrete causal inferences about the effect of weight stigma on engagement in physical health behaviours, and the mediating roles of stress and self-esteem. Very little research directly manipulates exposure to weight stigma in experimental designs and examines subsequent effects on physical health behaviours (see e.g. Incollingo Rodriguez et al., 2016; Kinkel-Ram et al., 2025; Lambert et al., 2019, for exceptions), and no research has used such designs to then examine stress or self-esteem as potential mediators. This may be a fruitful focus for further research. Additionally, there are a number of limitations in the measurement of key variables. All measures of behaviour were self-reported, which are prone to inaccuracies (Ravelli & Schoeller, 2020). Self-reported measures of weight and height were used, which are prone to inaccuracies. In addition, the measure of experienced weight stigma does not capture the frequency or intensity of exposure, limiting the ability to differentiate between individuals who have experienced repeated or chronic stigma and those who may have experienced it only once. A potential alternative method is ecological momentary assessment (EMA), which provides real-time data and may reduce biases associated with retrospective reporting. Future studies also could benefit from incorporating objective measures, particularly for PA (Godfrey et al., 2022). There were also limitations in the psychometric properties of some of the measures. Specifically, PSS-4 demonstrated relatively low internal consistency (*α* = .66), which may limit the precision with which stress is measured, potentially impacting observed relationships. Therefore, findings involving perceived stress should be interpreted with this constraint in mind. Relatedly, many of the questions included in the survey, though widely used in the literature (Gerend et al., 2023; Lee et al., 2021), have not undergone psychometric evaluation within the Indian population. This raises concerns regarding their cultural relevance, construct validity, and reliability in this context.

Lastly, as the study involved an English-language online survey, participation was limited to those who were fluent in English and had ready access to the internet. In addition, recruitment via social media and university networks meant that highly educated and digitally-literate young adults living in urban settings were overrepresented. The sample was also predominantly female. This limits the generalisability of the results to other samples. Future research should consider whether the relationship between weight stigma and physical health behaviours holds across samples with different demographic characteristics.

## Conclusion

The current study is the first to examine the association between weight stigma and engagement in physical health behaviours in an Indian context. Our findings closely mirror results reported in Western contexts, suggesting that the relationship between weight stigma and health behaviour presents similarly across cultures. The findings show that it is not only direct experiences of stigma that matter, but also the anticipation of being stigmatised and the internalisation of negative weight-related beliefs. The current study also lends empirical support to theoretical models that position stress as a key mechanism linking stigma to adverse health outcomes (Jackson et al., 2016; Tomiyama, 2014), through its impact on behaviour. Extending existing theory, our findings are also consistent with self-esteem acting an additional mediator, suggesting that stigma also undermines health behaviour by eroding individuals’ sense of self-worth. Overall, these findings highlight weight stigma as a potential barrier to engagement in healthy behaviour and suggest that interventions aimed at reducing weight stigma or disrupting its effect on stress and self-esteem may help improve health.

**Footnote:** The following variables were measured but analysis was not reported: Sleep duration, breakfast consumption, fast-food consumption, weight talk, weight loss behaviours by others, and social media usage. Sleep duration was excluded as it represented a health outcome rather than health behaviour. Breakfast and takeout/deliveries were excluded due to the difficulty in incontrovertibly classifying these behaviours as healthy or unhealthy. For transparency, the analyses for these variables are presented in the supplementary materials. The remaining variables were outside the scope of the current study. Further details are available on the OSF [pre-registration https://osf.io/3atdb/overview?view_only=6ee049f6f8774d45985c36d441e2ad5c; data and material https://osf.io/dax7b/overview].

## Supplementary Material

Supplementary Material Supplementary.docx

## Figures and Tables

**Table:** 

✔	(a) Institutional Review Board Statement: The study was conducted in accordance with the Declaration of Helsinki and was approved by an Institutional Review Board/Ethics committee. See details under Methods.
	**(b) The study received an exemption from an Institutional Review Board/Ethics committee**

## Data Availability

The data and materials that support the findings will be available in the Open Science Framework (OSF) at https://osf.io/dax7b/overview.

## Open scholarship

This article has earned the Centre for Open Science badges for Open Materials. The materials are openly accessible at https://osf.io/dax7b/overview; registration of study design and sampling plan of the larger project are openly accessible at https://osf.io/3atdb/overview?view_only=6ee049f6f8774d45985c36d441e2ad5c; registration of the hypotheses and analysis plan are openly accessible at https://osf.io/3atdb/overview?view_only=6ee049f6f8774d45985c36d441e2ad5c.
