# Supplementary material for: Weight stigma and engagement in physical health behaviours among young adults in India
Source: Health Psychol Behav Med. 2026 Jun 10;14(1):2687229. doi: 10.1080/21642850.2026.2687229 (PMC13255208; doi:10.1080/21642850.2026.2687229)
Supplement: Supplementary Material — Supplementary.docx [file RHPB_A_2687229_SM3732.docx]

**Weight Stigma and Engagement in Physical Health Behaviours among Young Adults in India**

**Supplementary Tables**

Table 1: Descriptive Statistics of Sample Characteristics and Key Study Variables

| Variable |  | Mean(SD) or n(%) |
| --- | --- | --- |
| Age |  | 19.93 (1.73) |
| Gender | Female | 622 (79.6%) |
|  | Male | 152 (19.5%) |
|  | Other identity | 2 (0.3%) |
|  | Prefer not to answer | 5 (0.6%) |
| Income | Less than INR 2,00,00 | 89 (11.4%) |
|  | INR 2,00,000 to 5,00,000 | 108 (13.4%) |
|  | INR 5,00,00 to 10,00,000 | 132 (16.9%) |
|  | INR 10,00,000 to 20,00,000 | 125 (16%) |
|  | INR 20,00,000 to 30,00,000 | 49 (6.3%) |
|  | INR 30,00,000 to 40,00,000 | 23 (2.9%) |
|  | INR 40,00,000 to 50,00,000 | 15 (1.9%) |
|  | INR 50,00,000 to 60,00,000 | 18(2.3%) |
|  | INR 60,00,000 to 70,00,000 | 11(1.4%) |
|  | INR 70,00,000 to 80,00,000 | 2 (.4%) |
|  | INR 80,00,000 to 90,00,000 | 11(21.4%) |
|  | Above INR 90,00,000 | 23 (2.9%) |
|  | Prefer not to answer | 173 (22.2%) |
| Education | Middle school or junior school | 6 (0.8%) |
|  | Some high school | 21 (2.7%) |
|  | High school graduate | 358 (45.8%) |
|  | Some college | 113 (14.5%) |
|  | Vocational, technical trade or certification program | 2 (0.3%) |
|  | Bachelor degree | 205 (26.2%) |
|  | Masters degree | 34 (4.4%) |
|  | Professional degree (MS, MBA, MD, PhD, etc) | 21(2.7%) |
|  | Other | 7 (0.9%) |
|  | Prefer not to answer | 12 (1.7%) |
| *BMI kg/m² | Underweight | 115 (16.20%) |
|  | Normal Weight | 401 (56.6%) |
|  | Overweight | 138 (19.4%) |
|  | Obese | 56 (7.8%) |
| Average BMI |  | 22.72 kg/m² |
| Weight Perception | Very underweight | 33 (4.6%) |
|  | Somewhat underweight | 111(15.5%) |
|  | About the right weight | 262 (36.5%) |
|  | Somewhat Overweight | 222(30.9%) |
|  | Very overweight | 90 (12.5%) |
| Binge eating without loss of control |  | 265 (33.9%) |
| Binge eating with loss of control |  | 179 (22.9%) |
| Unhealthy weight control behaviour | Fasted | 284 (37.5%) |
|  | Ate very little food | 379 (48.5%) |
|  | Skipped meals | 389 (49.8%) |
|  | Smoke more cigarettes | 62 (7.9%) |
|  | Used food substitutes | 94 (12%) |
| Extremely Unhealthy Weight Control Behaviour | Took diet pills | 43 (5.5%) |
|  | Throw up | 76 (9.7%) |
|  | Used Laxatives | 49 (6.27%) |
|  | Used diuretics | 35 (4.4%) |
| Physical Activity |  | 130.78 (143.99) |
| Breakfast | Never | 66 (8.6%) |
|  | 1-2 days | 135 (17.5%) |
|  | 3-4 days | 155 (20.1%) |
|  | 5-6 days | 85 (11%) |
|  | Everyday | 330 (42.8%) |
| Sleep on weekdays | Less than 7 hours | 191 (15.2%) |
|  | Between 7-9 hours | 325 (55.4%) |
|  | More than 9 hours | 71 (5.6%) |
| Sleep on weekends | Less than 7 hours | 46 (7.8%) |
|  | Between 7-9 hours | 334 (56.9%) |
|  | More than 9 hours | 206 (35.1%) |
| Alcohol Use |  | 1.57 (0.86) |
| Cigarettes |  | 1.48 (1.05) |
| E-Cigarettes |  | 1.35 (0.90) |
| Marijuana |  | 1.211 (0.63) |
| Drugs |  | 1.08 (0.46) |
| WBIS(M) |  | 2.74 (1.83) |
| AWS |  | 11.37 (3.82) |
| Experienced Weight Stigma | Teased | 351 (44.94%) |
|  | Treated Unfairly | 212 (27.14%) |
|  | Discriminated | 200 (25.6%) |
| Self Esteem |  | 17.72 (7.45) |
| Stress |  | 6.94 (3.82) |

**Note.** INR = Indian Rupee (1 INR = 0.011 USD); BMI = Body Mass Index; WBIS (M) = Modified Weight Bias Internalization Scale; AWS = Anticipated Weight Stigma; EWS = Experienced Weight Stigma. Although the total sample size was 781, some responses are shorter because participants were given the option not to answer questions they did not feel comfortable responding to. A total of 710 participants completed all weight stigma items, and 758 participants completed all health-related items.

*To ensure data quality, self-reported height and weight values were screened for implausible entries. Values falling outside expected adult ranges (height < 4 ft or > 7 ft; weight < 30 kg or > 250 kg) were flagged and cross-checked. No values outside these ranges were retained for analysis.

Table 2: Correlations Among Study Variables

|  | Age | BMI | Income | IWS | AWS | EWS | UWCB | EUWCB | HWCB | PA | Alcohol | Cig | E-Cig | Marijuana | Drugs | Breakfast | Sleep | SE | Stress |
| --- | --- | --- | --- | --- | --- | --- | --- | --- | --- | --- | --- | --- | --- | --- | --- | --- | --- | --- | --- |
| Age | 1 |  |  |  |  |  |  |  |  |  |  |  |  |  |  |  |  |  |  |
| BMI | .203^**^ | 1 |  |  |  |  |  |  |  |  |  |  |  |  |  |  |  |  |  |
| Income | 0.040 | -0.015 | 1 |  |  |  |  |  |  |  |  |  |  |  |  |  |  |  |  |
| IWS | 0.063 | .410^**^ | 0.021 | 1 |  |  |  |  |  |  |  |  |  |  |  |  |  |  |  |
| AWS | 0.054 | .386^**^ | 0.004 | .842^**^ | 1 |  |  |  |  |  |  |  |  |  |  |  |  |  |  |
| EWS | -0.003 | .201^**^ | 0.003 | .524^**^ | .595^**^ | 1 |  |  |  |  |  |  |  |  |  |  |  |  |  |
| UWCB | .116^**^ | .264^**^ | 0.005 | .332^**^ | .348^**^ | .216^**^ | 1 |  |  |  |  |  |  |  |  |  |  |  |  |
| EUWCB | .191^**^ | .198^**^ | -0.023 | .279^**^ | .278^**^ | .202^**^ | .506^**^ | 1 |  |  |  |  |  |  |  |  |  |  |  |
| HWCB | 0.014 | .243^**^ | 0.060 | .247^**^ | .196^**^ | .104^**^ | .219^**^ | -.085^*^ | 1 |  |  |  |  |  |  |  |  |  |  |
| PA | 0.003 | 0.032 | -0.034 | 0.014 | -0.071 | -0.055 | 0.050 | -0.013 | .360^**^ | 1 |  |  |  |  |  |  |  |  |  |
| Alcohol | .111^**^ | .147^**^ | .222^**^ | .157^**^ | .203^**^ | .128^**^ | .243^**^ | .210^**^ | 0.038 | 0.053 | 1 |  |  |  |  |  |  |  |  |
| Cig | .105^**^ | .164^**^ | .142^**^ | 0.065 | .076^*^ | .097^*^ | .250^**^ | .191^**^ | .076^*^ | .112^**^ | .619^**^ | 1 |  |  |  |  |  |  |  |
| E_Cig | 0.067 | .090^*^ | .198^**^ | 0.053 | 0.026 | 0.070 | .201^**^ | .227^**^ | 0.024 | .108^**^ | .611^**^ | .665^**^ | 1 |  |  |  |  |  |  |
| Marijuana | .088^*^ | .119^**^ | .097^*^ | 0.075 | 0.072 | 0.076 | .201^**^ | .221^**^ | -0.005 | .110^**^ | .560^**^ | .612^**^ | .606^**^ | 1 |  |  |  |  |  |
| Drugs | -0.017 | 0.024 | 0.015 | -0.003 | 0.031 | 0.066 | .146^**^ | .168^**^ | -0.045 | .117^**^ | .284^**^ | .332^**^ | .393^**^ | .513^**^ | 1 |  |  |  |  |
| Breakfast | -0.063 | -0.030 | -0.021 | -.075^*^ | -.078^*^ | -.087^*^ | -.242^**^ | -.152^**^ | .154^**^ | .073^*^ | -.112^**^ | -.140^**^ | -.209^**^ | -.123^**^ | -0.043 | 1 |  |  |  |
| Sleep | .033 | .057 | -.031 | .400** | .322** | .255** | .048 | .019 | .159** | .029 | .019 | -.054 | -.052 | -.005 | -.016 | .025 | 1 |  |  |
| SE | .083^*^ | -0.073 | 0.052 | .113^**^ | 0.025 | .162^**^ | -.108^**^ | -.107^**^ | .197^**^ | .138^**^ | -0.046 | -0.047 | -.080^*^ | -0.022 | -0.053 | .073^*^ | .505** | 1 |  |
| Stress | .085^*^ | .188^**^ | 0.030 | .614^**^ | .534^**^ | .379^**^ | .246^**^ | .282^**^ | .133^**^ | -0.063 | .149^**^ | 0.070 | .146^**^ | 0.063 | -0.035 | -.116^**^ | .464** | .262^**^ | 1 |
| n | 781 | 781 | 780 | 776 | 746 | 776 | 781 | 781 | 781 | 781 | 679 | 675 | 675 | 672 | 679 | 771 | 589 | 776 | 776 |

Note. **Correlation is significant at the 0.01 level (2-tailed) *Correlation is significant at the 0.05 level (2-tailed). IWS= Internalised Weight Stigma, AWS= Anticipated Weight Stigma, EWS= Experienced Weight Stigma, UWCB= Unhealthy weight control behaviour, EUWCB= Extremely unhealthy weight control behaviour, HWCB= Healthy weight control behaviour, PA= Physical activity, SE= Self-esteem

Table 3: Regression Coefficients of Experienced Weight Stigma on Health Behaviours Unadjusted and Adjusted for Age, Gender, BMI and Income

|  |  | ***Adjusted*** |  |  |  |  |  |  | ***Unadjusted*** |  |  |
| --- | --- | --- | --- | --- | --- | --- | --- | --- | --- | --- | --- |
|  |  | ***b*** | ***SE*** | ***β*** |  | **95% CI of *b*** | ***b*** | ***SE*** | ***β*** | ***p*** | **95% CI of *b*** |
| UWCB | EWS | 0.35 | 0.08 | 0.15 | **<.001** | [0.18, 0.51] | 0.49 | 0.08 | 0.22 | **<.001** | [0.33, 0.64] |
|  | Age | 0.07 | 0.02 | 0.10 | **.008** | [0.02, 0.11] |  |  |  |  |  |
|  | Gender | -0.08 | 0.09 | -0.03 | .383 | [-0.26,0.10] |  |  |  |  |  |
|  | BMI | 0.05 | 0.01 | 0.22 | **<.001** | [0.03, 0.07] |  |  |  |  |  |
|  | Income | 0.00 | 0.01 | 0.00 | .993 | [-0.02, 0.02] |  |  |  |  |  |
| EUWCB | EWS | 0.30 | 0.06 | 0.18 | **<.001** | [0.18, 0.42] | 0.06 | 0.01 | 0.20 | **<.001** | [0.04, 0.09] |
|  | Age | 0.09 | 0.02 | 0.19 | **<.001** | [0.06,0.13] |  |  |  |  |  |
|  | Gender | -0.07 | 0.07 | -0.04 | .320 | [-0.21, 0.07] |  |  |  |  |  |
|  | BMI | 0.02 | 0.01 | 0.13 | **<.001** | [0.01, 0.04] |  |  |  |  |  |
|  | Income | 0.00 | 0.01 | -0.02 | .495 | [-0.02,0.01] |  |  |  |  |  |
| HWCB | EWS | -0.46 | 0.40 | -0.04 | .245 | [-1.24, 0.32] | 1.30 | 0.45 | 0.10 | **.004** | [0.421, 2.183] |
|  | Age | -0.17 | 0.12 | -0.06 | .148 | [-0.40, 0.06] |  |  |  |  |  |
|  | Gender | 0.27 | 0.44 | 0.02 | .535 | [-0.59, 1.13] |  |  |  |  |  |
|  | BMI | 0.29 | 0.04 | 0.26 | **<.001** | [0.20, 0.37] |  |  |  |  |  |
|  | Income | 0.09 | 0.04 | 0.07 | .052 | [0.00, 0.17] |  |  |  |  |  |
| Physical Activity | EWS | -33.98 | 11.10 | -0.12 | **.002** | [-55.78,-12.19] | -15.86 | 10.35 | -0.05 | .126 | [4.47, -36.19] |
|  | Age | -2.39 | 3.27 | -0.03 | .465 | [-8.80,4.03] |  |  |  |  |  |
|  | Gender | 36.10 | 12.28 | 0.11 | **.003** | [11.98,60.22] |  |  |  |  |  |
|  | BMI | 1.36 | 1.19 | 0.04 | .256 | [-0.98,3.70] |  |  |  |  |  |
|  | Income | -2.52 | 1.23 | -0.08 | .**040** | [-4.93,-0.12] |  |  |  |  |  |
| Alcohol | EWS | 0.21 | 0.07 | 0.12 | **<.001** | [0.08, 0.34] | 0.22 | 0.07 | 0.13 | **<.001** | [0.09, 0.35] |
|  | Age | 0.04 | 0.02 | 0.08 | **.047** | [0.00, 0.08] |  |  |  |  |  |
|  | Gender | 0.16 | 0.07 | 0.09 | **.028** | [0.02, 0.30] |  |  |  |  |  |
|  | BMI | 0.02 | 0.01 | 0.10 | **.010** | [0.00, 0.03] |  |  |  |  |  |
|  | Income | 0.04 | 0.01 | 0.22 | **<.001** | [0.03, 0.06] |  |  |  |  |  |
| Cigarettes | EWS | 0.20 | 0.08 | 0.09 | **.014** | [0.04, 0.36] | 0.21 | 0.08 | 0.10 | **.011** | [0.05, 0.37] |
|  | Age | 0.03 | 0.02 | 0.05 | .214 | [-0.02, 0.08] |  |  |  |  |  |
|  | Gender | 0.40 | 0.09 | 0.17 | **<.001** | [0.22,0.58] |  |  |  |  |  |
|  | BMI | 0.03 | 0.01 | 0.12 | **.003** | [0.01, 0.04] |  |  |  |  |  |
|  | Income | 0.03 | 0.01 | 0.13 | **<.001** | [0.01, 0.05] |  |  |  |  |  |
| E-cigarettes | EWS | 0.13 | 0.07 | 0.07 | .062 | [-0.01, 0.27] | 0.13 | 0.07 | 0.07 | .069 | [-0.01, 0.27] |
|  | Age | 0.02 | 0.02 | 0.03 | .412 | [-0.02, 0.06] |  |  |  |  |  |
|  | Gender | 0.32 | 0.08 | 0.16 | **<.001** | [0.17, 0.47] |  |  |  |  |  |
|  | BMI | 0.01 | 0.01 | 0.05 | .175 | [0.00, 0.02] |  |  |  |  |  |
|  | Income | 0.04 | 0.01 | 0.19 | **<.001** | [0.02, 0.05] |  |  |  |  |  |
| Breakfast | EWS | -0.28 | 0.11 | -0.10 | **.011** | [-0.49, -0.06] | -0.24 | 0.10 | -0.09 | **.016** | [-0.44, -0.05] |
|  | Age | -0.05 | 0.03 | -0.06 | .135 | [-0.11, 0.01] |  |  |  |  |  |
|  | Gender | -0.15 | 0.12 | -0.05 | .212 | [-0.39, 0.09] |  |  |  |  |  |
|  | BMI | 0.00 | 0.01 | 0.01 | .888 | [-0.02, 0.02] |  |  |  |  |  |
|  | Income | 0.00 | 0.01 | -0.01 | .745 | [-0.03, 0.02] |  |  |  |  |  |
| Sleep | EWS | 0.91 | 0.24 | 0.14 | **<.001** | [0.43,1.38] | 1.79 | 0.24 | 0.25 | **<.001** | [1.31, 2.27] |
|  | Age | 0.04 | 0.07 | 0.02 | .611 | [-0.10, 0.18] |  |  |  |  |  |
|  | Gender | 0.52 | 0.27 | 0.08 | .055 | [-0.01, 1.04] |  |  |  |  |  |
|  | BMI | 0.01 | 0.03 | 0.01 | .762 | [-0.04, 0.06] |  |  |  |  |  |
|  | Income | -0.04 | 0.03 | -0.06 | .096 | [-0.01, 0.01] |  |  |  |  |  |

Note: Bold indicates statistical significance (*p* < .05). *SE* = standard error; *b* = unstandardised regression coefficient; *β =* standardised regression coefficient and *CI* confidence interval. BMI= Body Mass Index; EWS= Experienced Weight Stigma; UWCB= Unhealthy Weight Control Behaviour; EWUCB= Extremely Unhealthy Weight Control Behaviour; HWCB= Healthy Weight Control Behaviour

Table 4: Logistic Regression of Experienced Weight Stigma on Health Behaviours Unadjusted and Adjusted for Age, Gender, BMI and Income

|  |  | B | SE(B) | Wald | OR | Sig | 95% CI *B* | B | SE(B) | Wald | OR | Sig | 95% CI *B* |
| --- | --- | --- | --- | --- | --- | --- | --- | --- | --- | --- | --- | --- | --- |
| Binge Eating without loss of control | EWS | 0.58 | 0.17 | 11.65 | 1.78 | **<.001** | [1.28, 2.48] | 0.75 | 0.16 | 23.32 | 2.13 | **<.001** | [1.57, 2.89] |
|  | Age | 0.05 | 0.05 | 0.98 | 1.05 | .322 | [0.95, 1.15] |  |  |  |  |  |  |
|  | Gender | -0.14 | 0.19 | 0.55 | 0.87 | .459 | [0.60, 1.26] |  |  |  |  |  |  |
|  | BMI | 0.09 | 0.02 | 21.17 | 1.09 | **<.001** | [1.05, 1.14] |  |  |  |  |  |  |
|  | Income | 0.00 | 0.02 | 0.00 | 1.00 | .989 | [0.96, 1.04] |  |  |  |  |  |  |
| Binge Eating with loss of control | EWS | 0.62 | 0.19 | 10.12 | 1.85 | **.001** | [1.27, 2.72] | 0.96 | 0.18 | 28.97 | 2.60 | **<.001** | [1.84, 3.68] |
|  | Age | 0.10 | 0.05 | 3.70 | 0.10 | .055 | [1.00, 1.22] |  |  |  |  |  |  |
|  | Gender | -0.47 | 0.23 | 4.21 | -0.47 | **.040** | [0.40,0.98] |  |  |  |  |  |  |
|  | BMI | 0.10 | 0.02 | 23.24 | 0.10 | **<.001** | [1.06, 1.15] |  |  |  |  |  |  |
|  | Income | 0.02 | 0.02 | 0.55 | 0.02 | .457 | [0.97, 1.06] |  |  |  |  |  |  |

Note: Bold indicates statistical significance (*p* < .05). *SE* = standard error; *B* unstandardised regression coefficient and *CI* confidence interval. BMI= Body Mass Index; EWS= Experienced Weight Stigma; UWCB= Unhealthy Weight Control Behaviour; EWUCB= Extremely Unhealthy Weight Control Behaviour; HWCB= Healthy Weight Control Behaviour

Table 5: Regression Coefficients of Anticipated Weight Stigma on Health Behaviours, Unadjusted and Adjusted for Age, Gender, BMI and Income

|  |  |  | ***Adjusted*** |  |  |  |  | ***Unadjusted*** |  |  |  |
| --- | --- | --- | --- | --- | --- | --- | --- | --- | --- | --- | --- |
|  |  | ***b*** | ***SE*** | ***β*** | ***p*** | **95% CI of *b*** | ***b*** | ***SE*** | ***β*** | ***p*** | **95% CI of *b*** |
| UWCB | AWS | 0.04 | 0.01 | 0.29 | **<.001** | [0.03,0.05] | 0.05 | 0.00 | 0.35 | **<.001** | [0.04, 0.06] |
|  | Age | 0.07 | 0.02 | 0.10 | **.006** | [0.02,0.11] |  |  |  |  |  |
|  | Gender | -0.01 | 0.09 | 0.00 | .926 | [-0.19,0.17] |  |  |  |  |  |
|  | BMI | 0.03 | 0.01 | 0.14 | **<.001** | [0.02, 0.05] |  |  |  |  |  |
|  | Income | 0.00 | 0.01 | -0.01 | .722 | [-0.02, 0.01] |  |  |  |  |  |
| EUWCB | AWS | 0.06 | 0.01 | 0.18 | **<.001** | [0.04, 0.08] | 0.05 | 0.00 | 0.27 | **<.001** | [0.04, 0.07] |
|  | Age | 0.02 | 0.00 | 0.19 | **<.001** | [0.01, 0.03] |  |  |  |  |  |
|  | Gender | -0.01 | 0.01 | -0.04 | .281 | [-0.04, 0.01] |  |  |  |  |  |
|  | BMI | 0.00 | 0.00 | 0.12 | .001 | [0.00, 0.01] |  |  |  |  |  |
|  | Income | 0.00 | 0.00 | -0.03 | .478 | [0.00, 0.00] |  |  |  |  |  |
| HWCB | AWS | 0.01 | 0.03 | 0.01 | .768 | [-0.04, 0.06] | 0.14 | 0.03 | 0.20 | **<.001** | [0.09, 0.19] |
|  | Age | -0.15 | 0.12 | -0.05 | .191 | [-0.38,0.08] |  |  |  |  |  |
|  | Gender | 0.35 | 0.44 | 0.03 | .428 | [-0.52, 1.22] |  |  |  |  |  |
|  | BMI | 0.27 | 0.05 | 0.24 | **<.001** | [0.18, 0.36] |  |  |  |  |  |
|  | Income | 0.09 | 0.04 | 0.07 | .046 | [0.00,0.18] |  |  |  |  |  |
| Physical Activity | AWS | -2.51 | 0.72 | -0.14 | **<.001** | [-3.93, -1.09] | -1.22 | 0.63 | -0.07 | **.05** | [-2.45, .01] |
|  | Age | -1.82 | 3.26 | -0.02 | .576 | [-8.22, 4.58] |  |  |  |  |  |
|  | Gender | 32.72 | 12.38 | 0.10 | **.008** | [8.42, 57.02] |  |  |  |  |  |
|  | BMI | 2.25 | 1.27 | 0.07 | .077 | [-0.25, 4.75] |  |  |  |  |  |
|  | Income | -2.22 | 1.23 | -0.07 | .073 | [-4.64, 0.20] |  |  |  |  |  |
| Alcohol | AWS | 0.02 | 0.00 | 0.20 | **<.001** | [0.01, 0.03] | 0.02 | 0.00 | 0.20 | **<.001** | [0.01, 0.03] |
|  | Age | 0.04 | 0.02 | 0.07 | .055 | [0.00, 0.07] |  |  |  |  |  |
|  | Gender | 0.19 | 0.07 | 0.10 | **.009** | [0.05, 0.33] |  |  |  |  |  |
|  | BMI | 0.01 | 0.01 | 0.04 | .294 | [-0.01, 0.02] |  |  |  |  |  |
|  | Income | 0.04 | 0.01 | 0.22 | **<.001** | [0.03, 0.06] |  |  |  |  |  |
| Cigarettes | AWS | 0.01 | 0.01 | 0.05 | .212 | [0.00, 0.02] | 0.01 | 0.01 | 0.08 | **.049** | [0.00, 0.02] |
|  | Age | 0.03 | 0.02 | 0.05 | .251 | [-0.02, 0.08] |  |  |  |  |  |
|  | Gender | 0.39 | 0.09 | 0.17 | **<.001** | [0.21, 0.08] |  |  |  |  |  |
|  | BMI | 0.03 | 0.01 | 0.12 | **.006** | [0.01,0.04] |  |  |  |  |  |
|  | Income | 0.03 | 0.01 | 0.12 | **.001** | [0.01, 0.05] |  |  |  |  |  |
| E-cigarettes | AWS | 0.00 | 0.00 | 0.02 | .655 | [-0.01, 0.01] | 0.00 | 0.00 | 0.03 | .507 | [-0.01, 0.01] |
|  | Age | 0.02 | 0.02 | 0.03 | .431 | [-0.02,0.06] |  |  |  |  |  |
|  | Gender | 0.30 | 0.08 | 0.15 | **<.001** | [0.15, 0.46] |  |  |  |  |  |
|  | BMI | 0.01 | 0.01 | 0.06 | .152 | [0.00, 0.03] |  |  |  |  |  |
|  | Income | 0.04 | 0.01 | 0.19 | **<.001** | [0.02, 0.05] |  |  |  |  |  |
| Breakfast | AWS | -0.02 | 0.01 | -0.10 | **.022** | [-0.03, 0.00] | -0.01 | 0.01 | -0.08 | **.033** | [-0.02, 0.00] |
|  | Age | -0.05 | 0.03 | -0.06 | .153 | [-0.11, 0.02] |  |  |  |  |  |
|  | Gender | -0.16 | 0.12 | -0.05 | .176 | [-0.40, 0.07] |  |  |  |  |  |
|  | BMI | 0.01 | 0.01 | 0.02 | .676 | [-0.02, 0.03] |  |  |  |  |  |
|  | Income | 0.00 | 0.01 | -0.01 | .882 | [-0.03, 0.02] |  |  |  |  |  |
| Sleep | AWS | 0.10 | 0.02 | 0.27 | **<.001** | [0.07,0.13] | 0.13 | 0.01 | 0.32 | **<.001** | [0.10,0.16] |
|  | Age | 0.03 | 0.07 | 0.02 | .648 | [-0.10,0.17] |  |  |  |  |  |
|  | Gender | 0.64 | 0.26 | 0.09 | .015 | [0.12, 1.15] |  |  |  |  |  |
|  | BMI | -0.05 | 0.03 | -0.08 | .060 | [-0.10,0.00] |  |  |  |  |  |
|  | Income | -0.05 | 0.03 | -0.06 | .085 | [-0.10,0.01] |  |  |  |  |  |

Note: Bold indicates statistical significance (*p* < .05). *SE* = standard error; *b* = unstandardised regression coefficient; *β =* standardised regression coefficient; and *CI* confidence interval. BMI= Body Mass Index; AWS= Anticipated Weight Stigma; UWCB= Unhealthy Weight Control Behaviour; EWUCB= Extremely Unhealthy Weight Control Behaviour; HWCB= Healthy Weight Control Behaviour

Table 6: Logistic Regression of Anticipated Weight Stigma on Health Behaviours Unadjusted and Adjusted for Age, Gender, BMI and Income

| Adjusted | | | | | | | | Unadjusted | | | | | |
| --- | --- | --- | --- | --- | --- | --- | --- | --- | --- | --- | --- | --- | --- |
|  |  | B | SE(B) | Wald | OR | Sig | 95% CI *B* | B | SE(B) | Wald | OR | Sig | 95% CI *B* |
| Binge Eating without loss of control | AWS | 0.06 | 0.01 | 25.18 | 1.06 | **<.001** | [1.03,1.08] | 0.07 | 0.01 | 49.40 | 1.07 | **<.001** | [1.05, 1.09] |
|  | Age | 0.04 | 0.05 | 0.72 | 0.04 | .397 | [0.95, 1.15] |  |  |  |  |  |  |
|  | Gender | -0.04 | 0.19 | 0.05 | -0.04 | .827 | [0.66,1.39] |  |  |  |  |  |  |
|  | BMI | 0.06 | 0.02 | 9.65 | 0.06 | **.002** | [1.02, 1.11] |  |  |  |  |  |  |
|  | Income | 0.00 | 0.02 | 0.02 | 0.00 | .890 | [0.96, 1.04] |  |  |  |  |  |  |
| Binge Eating with loss of control | AWS | 0.06 | 0.01 | 23.49 | 1.06 | **<.001** | [1.04, 1.09] | 0.08 | 0.01 | 56.13 | 1.08 | **<.001** | [1.06, 1.11] |
|  | Age | 0.10 | 0.05 | 3.39 | 1.10 | .066 | [0.99,1.22] |  |  |  |  |  |  |
|  | Gender | -0.35 | 0.23 | 2.35 | 0.71 | .126 | [0.45, 1.10] |  |  |  |  |  |  |
|  | BMI | 0.07 | 0.02 | 10.77 | 1.07 | **.001** | [1.03, 1.12] |  |  |  |  |  |  |
|  | Income | 0.02 | 0.02 | 0.53 | 1.02 | .466 | [0.97 ,1.06] |  |  |  |  |  |  |

Note: Bold indicates statistical significance (*p* < .05). *SE* = standard error; *B* unstandardised regression coefficient and *CI* confidence interval. BMI= Body Mass Index; AWS= Anticipated Weight Stigma; UWCB= Unhealthy Weight Control Behaviour; EWUCB= Extremely Unhealthy Weight Control Behaviour; HWCB= Healthy Weight Control Behaviour

Table 7: Regression Coefficients of Internalised Weight Stigma on Health Behaviours Unadjusted and Adjusted for Age, Gender, BMI and Income

|  |  |  | ***Adjusted*** |  |  |  |  | ***Unadjusted*** | |  |  |  |
| --- | --- | --- | --- | --- | --- | --- | --- | --- | --- | --- | --- | --- |
|  |  | ***b*** | ***SE*** | ***β*** | ***p*** | **95% CI of *b*** | ***b*** | ***SE*** | ***β*** | | ***p*** | **95% CI of *b*** |
| UWCB | IWS | 0.02 | 0.00 | 0.27 | **<.001** | [0.01,0.02] | 0.02 | 0.00 | 0.33 | | **<.001** | [0.01, 0.02] |
|  | Age | 0.06 | 0.02 | 0.10 | **.008** | [0.02,0.12] |  |  |  | |  |  |
|  | Gender | -0.04 | 0.09 | -0.02 | .651 | [-0.22,0.14] |  |  |  | |  |  |
|  | BMI | 0.03 | 0.01 | 0.14 | **<.001** | [0.01,0.05] |  |  |  | |  |  |
|  | Income | 0.00 | 0.01 | -0.01 | .825 | [-0.02,0.02] |  |  |  | |  |  |
| EUWCB | IWS | 0.00 | 0.00 | 0.26 | **<.001** | [0.00,0.00] | 0.02 | 0.00 | 0.28 | | **<.001** | [0.02, 0.03] |
|  | Age | 0.02 | 0.00 | 0.19 | **<.001** | [0.01,0.03] |  |  |  | |  |  |
|  | Gender | -0.01 | 0.01 | -0.03 | .446 | [-0.04,0.02] |  |  |  | |  |  |
|  | BMI | 0.00 | 0.00 | 0.05 | .220 | [0.00,0.02] |  |  |  | |  |  |
|  | Income | 0.00 | 0.00 | -0.03 | .350 | [0.00,0.00] |  |  |  | |  |  |
| HWCB | IWS | 0.01 | 0.01 | 0.05 | .230 | [-0.01,0.03] | 0.07 | 0.01 | 0.25 | | **<.001** | [0.05, 0.09] |
|  | Age | -0.16 | 0.12 | -0.05 | .160 | [-0.39,0.06] |  |  |  | |  |  |
|  | Gender | 0.40 | 0.44 | 0.04 | .364 | [-0.46, 1.26] |  |  |  | |  |  |
|  | BMI | 0.25 | 0.05 | 0.23 | **<.001** | [0.16,0.34] |  |  |  | |  |  |
|  | Income | 0.08 | 0.04 | 0.07 | .059 | [0.00,0.17] |  |  |  | |  |  |
| Physical Activity | IWS | -0.56 | 0.30 | -0.08 | .060 | [-1.14,0.02] | 0.09 | 0.24 | 0.01 | | .701 | [-0.39,0.57] |
|  | Age | -2.17 | 3.28 | -0.03 | .509 | [-8.60, 3.27] |  |  |  | |  |  |
|  | Gender | 37.27 | 12.37 | 0.12 | **.003** | [12.99, 61.55] |  |  |  | |  |  |
|  | BMI | 1.58 | 1.29 | 0.05 | .219 | [-0.95,4.11] |  |  |  | |  |  |
|  | Income | -2.47 | 1.23 | -0.08 | **.046** | [-4.88,-0.05] |  |  |  | |  |  |
| Alcohol | IWS | 0.01 | 0.00 | 0.14 | **<.001** | [0.00, 0.01] | 0.01 | 0.00 | 0.16 | | **<.001** | [0.00, 0.01] |
|  | Age | 0.04 | 0.02 | 0.07 | .053 | [0.00,0.08] |  |  |  | |  |  |
|  | Gender | 0.17 | 0.07 | 0.09 | **.023** | [0.02,0.31] |  |  |  | |  |  |
|  | BMI | 0.01 | 0.01 | 0.07 | .119 | [0.00,0.03] |  |  |  | |  |  |
|  | Income | 0.04 | 0.01 | 0.22 | **<.001** | [0.03,0.06] |  |  |  | |  |  |
| Cigarettes | IWS | 0.00 | 0.00 | 0.03 | .433 | [0.00, 0.01] | 0.00 | 0.00 | 0.065 | | .088 | [0.00, 0.01] |
|  | Age | 0.03 | 0.02 | 0.05 | .239 | [-0.02,0.08] |  |  |  | |  |  |
|  | Gender | 0.39 | 0.09 | 0.17 | **<.001** | [0.21, 0.56] |  |  |  | |  |  |
|  | BMI | 0.03 | 0.01 | 0.13 | **.004** | [0.01,0.05] |  |  |  | |  |  |
|  | Income | 0.03 | 0.01 | 0.14 | **<.001** | [0.01,0.05] |  |  |  | |  |  |
| E-cigarettes | IWS | 0.00 | 0.00 | 0.06 | .142 | [0.00,0.01] | 0.00 | 0.00 | 0.05 | | .168 | [0.00, 0.01] |
|  | Age | 0.02 | 0.02 | 0.03 | .543 | [-0.02,0.06] |  |  |  | |  |  |
|  | Gender | 0.32 | 0.08 | 0.16 | **<.001** | [0.16, 0.47] |  |  |  | |  |  |
|  | BMI | 0.01 | 0.01 | 0.04 | .308 | [-0.01,0.02] |  |  |  | |  |  |
|  | Income | 0.04 | 0.01 | 0.19 | **<.001** | [0.02,0.05] |  |  |  | |  |  |
| Breakfast | IWS | -0.01 | 0.00 | -0.08 | .062 | [-0.01, .00] | 0.00 | 0.00 | -0.07 | | **.038** | [-0.01, .00] |
|  | Age | -0.05 | 0.03 | -0.06 | .101 | [-0.11, .02] |  |  |  | |  |  |
|  | Gender | -0.15 | 0.12 | -0.05 | .763 | [-0.38, .09] |  |  |  | |  |  |
|  | BMI | 0.01 | 0.01 | 0.02 | .737 | [-0.02, .03] |  |  |  | |  |  |
|  | Income | 0.00 | 0.01 | -0.01 | .667 | [-0.03, .02] |  |  |  | |  |  |
| Sleep | IWS | 0.05 | 0.01 | 0.31 | **<.001** | [0.04, 0.06] | 0.07 | 0.01 | 0.40 | | **<.001** | [0.06,0.08] |
|  | Age | 0.03 | 0.07 | 0.02 | .620 | [-0.10, 0.17] |  |  |  | |  |  |
|  | Gender | 0.67 | 0.26 | 0.10 | .010 | [0.16, 1.18] |  |  |  | |  |  |
|  | BMI | -0.06 | 0.03 | -0.09 | .028 | [-0.11,-0.01] |  |  |  | |  |  |
|  | Income | -0.05 | 0.03 | -0.07 | .048 | [-0.10,0.00] |  |  |  | |  |  |

Note: Bold indicates statistical significance (*p* < .05). *SE* = standard error; *b* = unstandardised regression coefficient; *β =* standardised regression coefficient; and *CI* confidence interval. BMI= Body Mass Index; IWS= Internalised Weight Stigma; UWCB= Unhealthy Weight Control Behaviour; EWUCB= Extremely Unhealthy Weight Control Behaviour; HWCB= Healthy Weight Control Behaviour

Table 8: Logistic Regression of Internalised Weight Stigma Health Behaviours Unadjusted and Adjusted for Age, Gender, BMI and Income

|  |  | B | SE(B) | Wald | OR | Sig | 95% CI of *b* | B | SE(B) | Wald | OR | Sig | 95% CI *b* |
| --- | --- | --- | --- | --- | --- | --- | --- | --- | --- | --- | --- | --- | --- |
| Binge Eating without loss of control | IWS | 0.02 | 0.00 | 27.07 | 1.02 | **<.001** | [1.01,1.03] | 0.03 | 0.00 | 50.40 | 1.03 | **<.001** | [1.02,1.04] |
|  | Age | 0.05 | 0.05 | 0.96 | 1.05 | .327 | [0.96,1.16] |  |  |  |  |  |  |
|  | Gender | -0.07 | 0.19 | 0.13 | 0.94 | .724 | [0.64,1.35] |  |  |  |  |  |  |
|  | BMI | 0.06 | 0.02 | 8.23 | 1.06 | **.004** | [1.02,1.10] |  |  |  |  |  |  |
|  | Income | 0.00 | 0.02 | 0.03 | 1.00 | .859 | [0.96,1.03] |  |  |  |  |  |  |
| Binge Eating with loss of control | IWS | 0.03 | 0.01 | 26.88 | 1.03 | **<.001** | [1.02,1.04] | 0.03 | 0.00 | 62.44 | 1.03 | **<.001** | [1.03,1.04] |
|  | Age | 0.10 | 0.05 | 3.72 | 1.11 | .051 | [1.00,1.23] |  |  |  |  |  |  |
|  | Gender | -0.37 | 0.23 | 2.63 | 0.69 | .051 | [0.44,1.08] |  |  |  |  |  |  |
|  | BMI | 0.07 | 0.02 | 9.47 | 1.07 | **.002** | [1.02,1.11] |  |  |  |  |  |  |
|  | Income | 0.01 | 0.02 | 0.31 | 1.01 | .695 | [0.97,1.06] |  |  |  |  |  |  |

Note: Bold indicates statistical significance (*p* < .05). *SE* = standard error; *B* unstandardised regression coefficient and *CI* confidence interval. BMI= Body Mass Index; IWS= Internalised Weight Stigma; UWCB= Unhealthy Weight Control Behaviour; EWUCB= Extremely Unhealthy Weight Control Behaviour; HWCB= Healthy Weight Control Behaviour

**Mediation Analysis**

Table 9 : Parallel Mediation Analyses of Experienced Weight Stigma on Health Behaviours via Self-Esteem and Perceived Stress

| **Path/Effect** | **Coefficient (Effect)** | **SE** | **t/Z** | **p-value** | **95% CI (LLCI, ULCI)** |
| --- | --- | --- | --- | --- | --- |
| UWCB |  |  |  |  |  |
| a1 (EWS → SE) | 0.3454 | 0.4964 | 0.696 | .4868 | [-0.6292, 1.3199] |
| a2 (EWS → Stress) | 1.6803 | 0.2505 | 6.707 | .0000 | **[1.1884, 2.1721]** |
| b1 (SE → UWCB) | -0.0346 | 0.0062 | -5.600 | .0000 | **[-0.0467, -0.0225]** |
| b2 (Stress → UWCB) | 0.0497 | 0.0122 | 4.056 | .0001 | **[0.0256, 0.0737]** |
| c (Total effect of EWS → UWCB) | 0.3477 | 0.0835 | 4.165 | .0000 | **[0.1838, 0.5115]** |
| c’ (Direct effect of EWS → UWCB) | 0.2761 | 0.0833 | 3.314 | .0010 | **[0.1125, 0.4397]** |
| Indirect effect (total) | 0.0715 | 0.0325 | — | — | **[0.0082, 0.1376]** |
| via SE | -0.0120 | 0.0176 | — | — | [-0.0492, 0.0218] |
| via Stress | 0.0835 | 0.0268 | — | — | **[0.0354, 0.1394]** |
| **EUWCB** |  |  |  |  |  |
| a1 (EWS → SE) | 0.3454 | 0.4964 | 0.696 | .4868 | [-0.6292, 1.3199] |
| a2 (EWS→ Stress) | 1.6803 | 0.2505 | 6.707 | .0000 | **[1.1884, 2.1721]** |
| b1(SE → EUWCB) | -0.0208 | 0.0046 | -4.504 | .0000 | [-0.0299, -0.0117] |
| b2(Stress → EUWCB) | 0.0605 | 0.0092 | 6.613 | .0000 | **[0.0426, 0.0785]** |
| c(Total effect of EWS → EUWCB) | 0.2999 | 0.0631 | 4.755 | .0000 | **[0.1761, 0.4238]** |
| c’(Direct effect of EWS → EUWCB) | 0.2054 | 0.0623 | 3.300 | .0010 | **[0.0832, 0.3277]** |
| Indirect effect (total) | 0.0945 | 0.0311 | — | — | **[0.0368, 0.1584]** |
| via SE | -0.0072 | 0.0110 | — | — | [-0.0314, 0.0130] |
| via Stress | 0.1017 | 0.0282 | — | — | **[0.0511, 0.1608]** |
| **PA** |  |  |  |  |  |
| a1 (EWS → SE) | 0.3454 | 0.4964 | 0.6958 | .4868 | [-0.6292, 1.3199] |
| a2 (EWS → Stress) | 1.6803 | 0.2505 | 6.7073 | <.0001 | **[1.1884, 2.1721]** |
| b1(SE → PA) | 0.7576 | 0.8379 | 0.9042 | .3662 | [-0.8875, 2.4028] |
| b2(Stress → PA) | -7.6768 | 1.6603 | -4.6239 | <.0001 | **[-10.9366, -4.4171]** |
| c(Total Effect of EWS → PA) | -33.9838 | 11.1003 | -3.0615 | .0023 | **[-55.7780, -12.1897]** |
| c’(Direct Effect (EWS → PA) | -21.3463 | 11.2959 | -1.8897 | .0592 | [-43.5246, 0.8320] |
| Indirect effect (total) | -12.6375 | 3.9119 | — | — | **[-20.8695, -5.5834]** |
| via SE | 0.2617 | 0.6795 | — | — | [-0.9208, 1.8871] |
| via Stress | -12.8992 | 3.7660 | — | — | **[-20.9014, -6.0929]** |
| **Alcohol** |  |  |  |  |  |
| a1 (EWS→ SE) | -1.1981 | 0.4290 | -2.7930 | .0054 | **[-2.0404, -0.3558]** |
| a2 (EWS→ Stress) | 1.0125 | 0.2336 | 4.3349 | <.0001 | **[0.5539, 1.4711]** |
| b1(SE → Alcohol) | 0.0035 | 0.0066 | 0.5289 | .5971 | [-0.0094, 0.0164] |
| b2(Stress → Alcohol) | 0.0361 | 0.0121 | 2.9938 | .0029 | **[0.0124, 0.0598]** |
| c(Total Effect of EWS → Alcohol) | 0.2128 | 0.0657 | 3.2367 | .0013 | **[0.0837, 0.3419]** |
| c’(Direct Effect (EWS→ Alcohol) | 0.1804 | 0.0664 | 2.7188 | .0067 | **[0.0501, 0.3107]** |
| Indirect effect (total) | 0.0324 | 0.0158 | — | — | **[0.0045, 0.0671]** |
| via SE | -0.0042 | 0.0079 | — | — | [-0.0208, 0.0106] |
| via Stress | 0.0366 | 0.0155 | — | — | **[0.0094, 0.0695]** |
| **Cigarettes** |  |  |  |  |  |
| a1 (EWS → SE) | -1.2342 | 0.4295 | -2.8736 | .0042 | **[-2.0776, -0.3909]** |
| a2 (EWS → Stress) | -0.0032 | 0.0082 | -0.3923 | .6950 | [-0.0193, 0.0129] |
| b1(SE → Cig) | 1.0349 | 0.2344 | 4.4158 | .0000 | **[0.5747, 1.4950]** |
| b2(Stress → Cig) | 0.0136 | 0.0150 | 0.9077 | .3644 | [-0.0159, 0.0432] |
| c(Total Effect of EWS→ Cig) | 0.2005 | 0.0817 | 2.4554 | .0143 | **[0.0402, 0.3608]** |
| c’(Direct Effect (EWS→ Cig) | 0.1824 | 0.0830 | 2.1987 | .0282 | **[0.0195, 0.3453]** |
| Indirect effect (total) | 0.0181 | 0.0162 | — | — | [-0.0116, 0.0530] |
| via SE | 0.0040 | 0.0104 | — | — | [-0.0153, 0.0272] |
| via Stress | 0.0141 | 0.0138 | — | — | [-0.0122, 0.0438] |
| **Breakfast** |  |  |  |  |  |
| a1 (EWS → SE) | 0.3454 | 0.4964 | 0.6958 | .4868 | [-0.6292, 1.3199] |
| a2 (EWS → Stress) | 0.0235 | 0.0082 | 2.8523 | .0045 | **[0.0073, 0.0397]** |
| b1(SE → Breakfast) | 1.6803 | 0.2505 | 6.7073 | .0000 | **[1.1884, 2.1721]** |
| b2(Stress → Breakfast) | -0.0449 | 0.0163 | -2.7513 | .0061 | [-0.0769, -0.0129] |
| c(Total Effect of EWS → Breakfast) | -0.2762 | 0.1086 | -2.5427 | .0112 | [-0.4895, -0.0629] |
| c’(Direct Effect (EWS → Breakfast) | -0.2089 | 0.1110 | -1.8816 | .0603 | [-0.4269, 0.0091] |
| Indirect effect (total) | -0.0673 | 0.0329 | — | — | [-0.1333, -0.0036] |
| via SE | 0.0081 | 0.0127 | — | — | [-0.0154, 0.0368] |
| via Stress | -0.0754 | 0.0301 | — | — | **[-0.1367, -0.0179]** |
| **Sleep** |  |  |  |  |  |
| a1 (EWS → SE) | 0.3454 | 0.4964 | 0.6958 | 0.4868 | [-0.6292, 1.3199] |
| a2 (EWS → Stress) | 1.6803 | 0.2505 | 6.7073 | .0000 | **[1.1884, 2.1721]** |
| b1(SE → **Sleep**) | 0.1783 | 0.0166 | 10.7404 | .0000 | **[0.1457, 0.2109]** |
| b2(Stress → **Sleep**) | 0.2854 | 0.0329 | 8.6784 | .0000 | **[0.2209, 0.3500]** |
| c(Total Effect of EWS → **Sleep**) | 0.9076 | 0.2429 | 3.7358 | 0.0002 | **[0.4306, 1.3845]** |
| c’(Direct Effect (EWS → **Sleep**) | 0.3664 | 0.2238 | 1.6371 | 0.1021 | [-0.0730, 0.8057] |
| Indirect effect (total) | 0.5412 | 0.1271 | — | — | **[0.3078, 0.8045]** |
| via SE | 0.0616 | 0.0897 | — | — | [-0.1088, 0.2469] |
| via Stress | 0.4796 | 0.0963 | — | — | **[0.3027, 0.6887]** |
| **Binge Eating without loss of control** |  |  |  |  |  |
| EWS → SE | 0.3327 | 0.4971 | 0.6694 | .5035 | [-0.6433, 1.3087] |
| EWS→ Stress | 1.6797 | 0.2509 | 6.6939 | .0000 | **[1.1871, 2.1724]** |
| SE → OE | -0.0820 | 0.0139 | -5.8923 | .0000 | [-0.1092, -0.0547] |
| Stress → OE | 0.0902 | 0.0266 | 3.3910 | .0007 | **[0.0381, 0.1424]** |
| Direct effect: EWS → OE | 0.5033 | 0.1812 | 2.7776 | .0055 | **[0.1481, 0.8584]** |
| Total indirect effect | -0.0273 | 0.0423 | — | — | [-0.1138, 0.0529] |
| Indirect effect via SE | 0.1516 | 0.0498 | — | — | **[0.0662, 0.2611]** |
| Indirect effect via Stress | 0.1243 | 0.0666 | — | — | **[0.0010, 0.2631]** |
| **Binge Eating with loss of control** |  |  |  |  |  |
| EWS→ SE | 0.3454 | 0.4964 | 0.696 | .487 | [-0.6292, 1.3199] |
| EWS→ Stress | 1.6803 | 0.2505 | 6.707 | <.001 | **[1.1884, 2.1721]** |
| SE → BE | -0.0986 (log-odds) | 0.0157 | -6.281 | <.001 | **[-0.1293, -0.0678]** |
| Stress → BE | 0.0715 (log-odds) | 0.0288 | 2.483 | .013 | **[0.0151, 0.1279]** |
| Direct effect (EWS → BE) | 0.5954 (log-odds) | 0.2123 | 2.805 | .005 | **[0.1793, 1.0115]** |
| Total indirect effect | -0.0340 | 0.0511 | — | — | [-0.1414, 0.0615] |
| Indirect effect via SE | 0.1201 | 0.0551 | — | — | **[0.0226, 0.2383]** |
| Indirect effect via Stress | 0.0861 | 0.0765 | — | — | [-0.0598, 0.2393] |

**Note.** Age, gender and BMI were adjusted for the model. Significant effects (p < .05 or 95% CI not containing zero) are **bolded**. Bootstrapped 95% confidence intervals (in parentheses) are based on 5000 samples. EWS= Experienced Weight Stigma; SE= Self-esteem; UWCB= Unhealthy Weight Control Behaviour; EWUCB= Extremely Unhealthy Weight Control Behaviour; PA= Physical Activity

Table 10 : Parallel Mediation Analyses of Anticipated Weight Stigma on Health Behaviours via Self-Esteem and Perceived Stress

| **Path/Effect** | **Coefficient (Effect)** | **SE** | **t** | **p-value** | **95% CI (LLCI, ULCI)** |
| --- | --- | --- | --- | --- | --- |
| a1 (AWS → SE) | -0.1129 | 0.0316 | -3.578 | .0004 | **[-0.1749, -0.0509]** |
| a2 (AWS → Stress) | 0.1817 | 0.0151 | 12.071 | .0000 | **[0.1522, 0.2113]** |
| b1 (SE → UWCB) | -0.0282 | 0.0063 | -4.470 | .0000 | **[-0.0405, -0.0158]** |
| b2 (Stress → UWCB) | 0.0346 | 0.0132 | 2.617 | .0091 | **[0.0086, 0.0605]** |
| c (Total effect of AWS → UWCB) | 0.0393 | 0.0053 | 7.431 | .0000 | **[0.0289, 0.0497]** |
| c’ (Direct effect of AWS → UWCB) | 0.0299 | 0.0058 | 5.189 | .0000 | **[0.0186, 0.0412]** |
| Indirect effect (total) | 0.0095 | 0.0029 | — | — | **[0.0036, 0.0151]** |
| via SE | 0.0032 | 0.0013 | — | — | **[0.0009, 0.0059]** |
| via Stress | 0.0063 | 0.0027 | — | — | **[0.0011, 0.0117]** |
| **EUWCB** |  |  |  |  |  |
| a1 (AWS → SE) | -0.1129 | 0.0316 | -3.578 | .0004 | **[-0.1749, -0.0509]** |
| a2 (AWS → Stress) | 0.1817 | 0.0151 | 12.071 | .0000 | **[0.1522, 0.2113]** |
| b1(SE → EUWCB) | -0.0182 | 0.0048 | -3.795 | .0002 | **[-0.0277, -0.0088]** |
| b2(Stress → EUWCB) | 0.0545 | 0.0101 | 5.409 | .0000 | **[0.0347, 0.0743]** |
| c(Total effect of AWS → EUWCB) | 0.0270 | 0.0041 | 6.609 | .0000 | **[0.0190, 0.0350]** |
| c’(Direct effect of AWS → EUWCB) | 0.0151 | 0.0044 | 3.428 | .0006 | **[0.0064, 0.0237]** |
| Indirect effect (total) | 0.0120 | 0.0027 | — | — | **[0.0069, 0.0171]** |
| via SE | 0.0021 | 0.0008 | — | — | **[0.0006, 0.0038]** |
| via Stress | 0.0099 | 0.0026 | — | — | **[0.0051, 0.0151]** |
| **PA** |  |  |  |  |  |
| a1 (AWS → SE) | -0.1129 | 0.0316 | -3.5780 | .0004 | **[-0.1749, -0.0509]** |
| a2 (AWS → Stress) | 0.1817 | 0.0151 | 12.0705 | .0000 | **[0.1522, 0.2113]** |
| b1(SE → PA) | 0.4997 | 0.8668 | 0.5764 | .5645 | [-1.2023, 2.2016] |
| b2(Stress → PA) | -7.5920 | 1.8170 | -4.1784 | .0000 | **[-11.1595, -4.0245]** |
| c(Total Effect of AWS → PA) | -2.5054 | 0.7232 | -3.4642 | .0006 | **[-3.9254, -1.0854]** |
| c’(Direct Effect (AWS → PA) | -1.0694 | 0.7921 | -1.3502 | .1774 | [-2.6246, 0.4858] |
| Indirect effect (total) | -1.4360 | 0.3835 | — | — | **[-2.1938, -0.7277]** |
| via SE | -0.0564 | 0.1208 | — | — | [-0.3217, 0.1747] |
| via Stress | -1.3796 | 0.3812 | — | — | **[-2.1370, -0.6554]** |
| **Alcohol** |  |  |  |  |  |
| a1 (AWS → SE) | -0.2592 | 0.0269 | -9.6486 | .0000 | **[-0.3120, -0.2065]** |
| a2 (AWS → Stress) | 0.1324 | 0.0148 | 8.9613 | .0000 | **[0.1034, 0.1615]** |
| b1(SE → Alcohol) | 0.0098 | 0.0069 | 1.4350 | .1518 | [-0.0036, 0.0233] |
| b2(Stress → Alcohol) | 0.0272 | 0.0125 | 2.1802 | .0296 | **[0.0027, 0.0517]** |
| c(Total Effect of AWS → Alcohol) | 0.0220 | 0.0044 | 5.0366 | .0000 | **[0.0134, 0.0306]** |
| c’(Direct Effect (AWS→ Alcohol) | 0.0209 | 0.0048 | 4.3999 | .0000 | **[0.0116, 0.0303]** |
| Indirect effect (total) | 0.0010 | 0.0022 | — | — | [-0.0035, 0.0054] |
| via SE | -0.0026 | 0.0017 | — | — | [-0.0063, 0.0007] |
| via Stress | 0.0036 | 0.0018 | — | — | **[0.0003, 0.0073]** |
| **Breakfast** |  |  |  |  |  |
| a1 (AWS → SE) | -0.1129 | 0.0316 | -3.5780 | .0004 | **[-0.1749, -0.0509]** |
| a2 (AWS → Stress) | 0.1817 | 0.0151 | 12.0705 | .0000 | **[0.1522, 0.2113]** |
| b1(SE → Breakfast) | 0.0216 | 0.0085 | 2.5298 | .0116 | **[0.0048, 0.0383]** |
| b2(Stress → Breakfast) | -0.0476 | 0.0179 | -2.6637 | .0079 | **[-0.0827, -0.0125]** |
| c(Total Effect of AWS → Breakfast) | -0.0163 | 0.0071 | -2.2943 | .0221 | **[-0.0302, -0.0023]** |
| c’(Direct Effect (AWS → Breakfast) | -0.0052 | 0.0078 | -0.6660 | .5056 | [-0.0205, 0.0101] |
| Indirect effect (total) | -0.0111 | 0.0036 | — | — | **[-0.0183, -0.0045]** |
| via SE | -0.0024 | 0.0013 | — | — | **[-0.0054, -0.0003]** |
| via Stress | -0.0087 | 0.0035 | — | — | **[-0.0157, -0.0020]** |
| **Sleep** |  |  |  |  |  |
| a1 (AWS → SE) | -0.1129 | 0.0316 | -3.578 | 0.0004 | **[-0.1749, -0.0509]** |
| a2 (AWS → Stress) | 0.1817 | 0.0151 | 12.0705 | 0.0000 | **[0.1522, 0.2113]** |
| b1(SE → Sleep) | 0.1866 | 0.0168 | 11.1042 | 0.0000 | **[0.1536, 0.2196]** |
| b2(Stress → Sleep) | 0.2073 | 0.0352 | 5.8848 | 0.0000 | **[0.1381, 0.2765]** |
| c(Total Effect of AWS → Sleep) | 0.1023 | 0.0153 | 6.6788 | 0.0000 | **[0.0722, 0.1324]** |
| c’(Direct Effect (AWS → Sleep) | 0.0857 | 0.0154 | 5.5813 | 0.0000 | **[0.0556, 0.1159]** |
| Indirect effect (total) | 0.0166 | 0.0113 | — | — | [-0.0049, 0.0393] |
| via SE | -0.0211 | 0.0073 | — | — | **[-0.0353, -0.0063]** |
| via Stress | 0.0377 | 0.0076 | — | — | **[0.0234, 0.0536]** |
| **Binge Eating without loss of control** |  |  |  |  |  |
| AWS → SE | -0.1131 | 0.0316 | -3.5823 | .0004 | **[-0.1751, -0.0511]** |
| AWS → Stress | 0.1817 | 0.0151 | 12.0594 | .0000 | **[0.1521, 0.2113]** |
| SE → OE | -0.0777 | 0.0142 | -5.4626 | .0000 | **[-0.1056, -0.0498]** |
| Stress → OE | 0.0643 | 0.0289 | 2.2238 | .0262 | **[0.0076, 0.1211]** |
| Direct effect: AWS → OE | 0.0382 | 0.0123 | 3.0978 | .0019 | **[0.0140, 0.0624]** |
| Total indirect effect | 0.0205 | 0.0064 | — | — | **[0.0086, 0.0339]** |
| Indirect effect via SE | 0.0088 | 0.0035 | — | — | **[0.0025, 0.0165]** |
| Indirect effect via Stress | 0.0117 | 0.0054 | — | — | **[0.0018, 0.0230]** |
| **Binge Eating with loss of control** |  |  |  |  |  |
| AWS → SE | -0.1129 | 0.0316 | -3.5780 | .0004 | **[-0.1749, -0.0509]** |
| AWS → Stress | 0.1817 | 0.0151 | 12.0705 | .0000 | **[0.1522, 0.2113]** |
| SE → BE | -0.0920 | 0.0160 | -5.7648 | .0000 | **[-0.1233, -0.0607]** |
| Stress → BE | 0.0432 | 0.0318 | 1.3594 | .1740 | [-0.0191, 0.1055] |
| Direct effect (AWS → BE) | 0.0433 | 0.0136 | 3.1826 | .0015 | **[0.0166, 0.0700]** |
| Total indirect effect | 0.0182 | 0.0076 | — | — | **[0.0045, 0.0342]** |
| Indirect effect via SE | 0.0104 | 0.0041 | — | — | **[0.0030, 0.0190]** |
| Indirect effect via Stress | 0.0079 | 0.0063 | — | — | [-0.0040, 0.0213] |

**Note.** Age, gender and BMI were adjusted for the model. Significant effects (p < .05 or 95% CI not containing zero) are **bolded**. Bootstrapped 95% confidence intervals (in parentheses) are based on 5000 samples. AWS= Anticipated Weight Stigma; SE= Self-esteem; UWCB= Unhealthy Weight Control Behaviour; EWUCB= Extremely Unhealthy Weight Control Behaviour; PA= Physical Activity

Table 11 : Parallel Mediation Analyses of Internalised Weight Stigma on Health Behaviours via Self-Esteem and Perceived Stress

| Path/Effect | Coefficient (Effect) | SE (Bootstrapping SE) | t/z | p-value | 95% CI (LLCI, ULCI) |
| --- | --- | --- | --- | --- | --- |
| **UWCB** |  |  |  |  |  |
| a1 (IWS → SE) | -0.048 | 0.013 | -3.659 | .000 | **[-0.073, -0.022]** |
| a2 (IWS → Stress) | 0.088 | 0.006 | 14.704 | .000 | **[ 0.076, 0.100]** |
| b1 (SE → UWCB) | -0.030 | 0.006 | -4.891 | .000 | **[-0.042, -0.018]** |
| b2 (Stress → UWCB) | 0.031 | 0.013 | 2.301 | .022 | **[ 0.005, 0.058]** |
| c (Total effect of IWS→ UWCB) | 0.015 | 0.002 | 6.911 | .000 | **[ 0.011, 0.019]** |
| c’ (Direct effect of IWS → UWCB) | 0.011 | 0.003 | 4.399 | .000 | **[ 0.006, 0.016]** |
| Indirect effect (total) | 0.004 | 0.001 | — | — | **[ 0.001, 0.007]** |
| via SE | 0.001 | 0.001 | — | — | **[ 0.000, 0.003]** |
| via Stress | 0.003 | 0.001 | — | — | **[ 0.000, 0.005]** |
| **EUWCB** |  |  |  |  |  |
| a1 (IWS → SE) | -0.0478 | 0.0131 | -3.659 | .0003 | **[-0.0734, -0.0221]** |
| a2 (IWS → Stress) | 0.0881 | 0.0060 | 14.704 | .0000 | **[0.0764, 0.0999]** |
| b1(SE → EUWCB) | -0.0186 | 0.0047 | -3.988 | .0001 | **[-0.0277, -0.0094]** |
| b2(Stress → EUWCB) | 0.0539 | 0.0102 | 5.311 | .0000 | **[0.0340, 0.0739]** |
| c(Total effect of IWS → EUWCB) | 0.0109 | 0.0017 | 6.578 | .0000 | **[0.0076, 0.0141]** |
| c’(Direct effect of IWS → EUWCB) | 0.0052 | 0.0019 | 2.818 | .0050 | **[0.0016, 0.0089]** |
| Indirect effect (total) | 0.0056 | 0.0013 | — | — | **[0.0032, 0.0082]** |
| via SE | 0.0009 | 0.0003 | — | — | **[0.0003, 0.0016]** |
| via Stress | 0.0048 | 0.0012 | — | — | **[0.0024, 0.0073]** |
| **Alcohol** |  |  |  |  |  |
| a1 (IWS → SE) | -0.1239 | 0.0108 | -11.426 | .0000 | **[-0.1452, -0.1026]** |
| a2 (IWS → Stress) | 0.0654 | 0.0060 | 10.895 | .0000 | **[0.0536, 0.0772]** |
| b1(SE → Alcohol) | 0.0076 | 0.0069 | 1.103 | .2705 | [-0.0059, 0.0210] |
| b2(Stress → Alcohol) | 0.0326 | 0.0124 | 2.628 | .0088 | **[0.0082, 0.0569]** |
| c(Total Effect of IWS → Alcohol) | 0.0062 | 0.0018 | 3.427 | .0006 | **[0.0026, 0.0097]** |
| c’(Direct Effect (IWS → Alcohol) | 0.0050 | 0.0020 | 2.449 | .0146 | **[0.0010, 0.0090]** |
| Indirect effect (total) | 0.0012 | 0.0012 | — | — | [-0.0012, 0.0035] |
| via SE | -0.0009 | 0.0009 | — | — | [-0.0027, 0.0007] |
| via Stress | 0.0021 | 0.0009 | — | — | **[0.0004, 0.0039]** |
| **Breakfast** |  |  |  |  |  |
| a1 (IWS → SE) | -0.0482 | 0.0131 | -3.689 | .0002 | **[-0.0739, -0.0226]** |
| a2 (IWS → Stress) | 0.0881 | 0.0060 | 14.672 | .0000 | **[0.0763, 0.0999]** |
| b1(SE → Breakfast) | 0.0229 | 0.0083 | 2.747 | .0062 | **[0.0065, 0.0392]** |
| b2(Stress → Breakfast) | -0.0524 | 0.0181 | -2.893 | .0039 | **[-0.0880, -0.0168]** |
| c(Total Effect of IWS → Breakfast) | -0.0058 | 0.0029 | -1.991 | .0468 | **[-0.0114, -0.0001]** |
| c’(Direct Effect (IWS → Breakfast) | 0.0000 | 0.0033 | -0.013 | .9899 | [-0.0065, 0.0065] |
| Indirect effect (total) | -0.0057 | 0.0017 | — | — | **[-0.0092, -0.0024]** |
| via SE | -0.0011 | 0.0006 | — | — | **[-0.0024, -0.0002]** |
| via Stress | -0.0046 | 0.0017 | — | — | **[-0.0081, -0.0014]** |
| **Sleep** |  |  |  |  |  |
| a1 (IWS → SE) | -0.0482 | 0.0131 | -3.6891 | 0.0002 | **[-0.0739, -0.0226]** |
| a2 (IWS → Stress) | 0.0881 | 0.006 | 14.6723 | 0 | **[0.0763, 0.0999]** |
| b1(SE → Sleep) | 0.193 | 0.0163 | 11.8483 | 0 | **[0.1610, 0.2250]** |
| b2(Stress → Sleep) | 0.1889 | 0.0355 | 5.3264 | 0 | **[0.1193, 0.2585]** |
| c(Total Effect of IWS → Sleep) | 0.0487 | 0.0063 | 7.7887 | 0 | **[0.0364, 0.0610]** |
| c’(Direct Effect (IWS → Sleep) | 0.0414 | 0.0065 | 6.3914 | 0 | **[0.0287, 0.0541]** |
| Indirect effect (total) | 0.0073 | 0.0054 | — | — | **[-0.0025, 0.0185]** |
| via SE | -0.0093 | 0.0032 | — | — | **[-0.0156, -0.0030]** |
| via Stress | 0.0166 | 0.0038 | — | — | **[0.0097, 0.0246]** |
| **Binge Eating without loss of control** |  |  |  |  |  |
| IWS → SE | -0.0482 | 0.0131 | -3.683 | .0002 | **[-0.0738, -0.0225]** |
| IWS → Stress | 0.0881 | 0.0060 | 14.667 | .0000 | **[0.0763, 0.0999]** |
| SE → OE | -0.0753 | 0.0140 | -5.390 | .0000 | **[-0.1027, -0.0479]** |
| Stress → OE | 0.0668 | 0.0294 | 2.274 | .0230 | **[0.0092, 0.1243]** |
| Direct effect: IWS → OE | 0.0153 | 0.0052 | 2.927 | .0034 | **[0.0051, 0.0256]** |
| Total indirect effect | 0.0095 | 0.0029 | — | — | **[0.0039, 0.0154]** |
| Indirect effect via SE | 0.0036 | 0.0014 | — | — | **[0.0011, 0.0066]** |
| Indirect effect via Stress | 0.0059 | 0.0026 | — | — | **[0.0011, 0.0113]** |
| **Binge Eating with loss of control** |  |  |  |  |  |
| IWS → SE | -0.0482 | 0.0131 | -3.689 | .0002 | **[-0.0739, -0.0226]** |
| IWS → Stress | 0.0881 | 0.0060 | 14.672 | .0000 | **[0.0763, 0.0999]** |
| SE → BE | -0.0907 | 0.0159 | -5.723 | .0000 | **[-0.1218, -0.0597]** |
| Stress → BE | 0.0404 | 0.0324 | 1.244 | .2133 | [-0.0232, 0.1039] |
| Direct effect (IWS→ BE) | 0.0189 | 0.0058 | 3.272 | .0011 | **[0.0076, 0.0302]** |
| Total indirect effect | 0.0079 | 0.0036 | — | — | **[0.0010, 0.0150]** |
| Indirect effect via SE | 0.0044 | 0.0017 | — | — | **[0.0014, 0.0078]** |
| Indirect effect via Stress | 0.0036 | 0.0031 | — | — | [-0.0024, 0.0098] |

**Note.** Age, gender and BMI were adjusted for the model. Significant effects (p < .05 or 95% CI not containing zero) are **bolded**. Bootstrapped 95% confidence intervals (in parentheses) are based on 5000 samples. IWS=: Internalised Weight Stigma; SE= Self-esteem; UWCB= Unhealthy Weight Control Behaviour; EWUCB= Extremely Unhealthy Weight Control Behaviour

| Table 12: Moderation of the Association Between Experienced Weight Stigma and Health Behaviour by Weight Perception | | | | | | | |
| --- | --- | --- | --- | --- | --- | --- | --- |
|  |  | *b* | *SE* | *t* | *p* | | 95% CI of *b* |
| UWCB | EWS | -0.26 | 0.29 | -0.90 | .37 | | [-0.83, 0.30] |
|  | Weight Perception | 0.14 | 0.07 | 1.93 | **.05** | | [-0.00, 0.29] |
|  | Weight Perception x EWS | 0.18 | 0.09 | 2.13 | **.03** | | [0.01, 0.35] |
|  | Age | 0.07 | 0.02 | 3.02 | **.00** | | [0.03, 0.12] |
|  | Gender | -0.01 | 0.09 | -0.12 | .91 | | [-0.19, 0.17] |
|  | BMI | 0.01 | 0.01 | 0.72 | .47 | | [-0.02, 0.03] |
|  | Income | -0.00 | 0.01 | -0.14 | .89 | | [-0.02, 0.02] |
| EUWCB | EWS | -0.42 | 0.22 | -1.92 | .06 | | [-0.85, 0.01] |
|  | Weight Perception | -0.05 | 0.06 | -0.82 | .41 | | [-0.16, 0.06] |
|  | Weight Perception x EWS | 0.22 | 0.07 | 3.40 | **< .001** | [0.09, 0.35] | |
|  | Age | 0.10 | 0.02 | 5.35 | **< .001** | | [0.06, 0.14] |
|  | Gender | -0.05 | 0.07 | -0.68 | .49 | | [-0.19, 0.09] |
|  | BMI | 0.01 | 0.01 | 0.54 | .59 | | [-0.01, 0.02] |
|  | Income | -0.01 | 0.01 | -0.79 | .43 | | [-0.02, 0.01] |
| HWCB | EWS | 0.46 | 1.38 | 0.33 | .74 | | [-2.25, 3.17] |
|  | Weight Perception | 0.95 | 0.36 | 2.66 | **.008** | | [0.25, 1.65] |
|  | Weight Perception x EWS | -0.30 | 0.41 | -0.74 | .46 | | [-1.11, 0.50] |
|  | Age | -0.15 | 0.12 | -1.31 | .19 | | [-0.38, 0.08] |
|  | Gender | 0.50 | 0.44 | 1.12 | .26 | | [-0.37, 1.37] |
|  | BMI | 0.18 | 0.06 | 2.94 | **.003** | | [0.06, 0.29] |
|  | Income | 0.08 | 0.04 | 1.89 | .06 | | [-0.00, 0.17] |
| Physical Activity | EWS | -30.53 | 38.80 | -0.79 | .432 | | [-106.70, 45.64] |
|  | Weight Perception | -15.65 | 10.01 | -1.56 | .118 | | [-35.31, 4.00] |
|  | Weight Perception x EWS | -0.72 | 11.51 | -0.06 | .950 | | [-23.33, 21.89] |
|  | Age | -2.85 | 3.27 | -0.87 | .384 | | [-9.27, 3.57] |
|  | Gender | 31.43 | 12.45 | 2.52 | **.012** | | [6.99, 55.87] |
|  | BMI | 3.82 | 1.69 | 2.26 | **.024** | | [0.51, 7.14] |
|  | Income | -2.46 | 1.22 | -2.01 | .045 | | [-4.86, -0.06] |
| Alcohol Use | EWS | 0.32 | 0.24 | 1.34 | .180 | | [-0.15, 0.78] |
|  | Weight Perception | 0.15 | 0.06 | 2.40 | **.017** | | [0.03, 0.27] |
|  | Weight Perception x EWS | -0.04 | 0.07 | -0.51 | .608 | | [-0.17, 0.10] |
|  | Age | 0.04 | 0.02 | 2.08 | **.038** | | [0.00, 0.08] |
|  | Gender | 0.20 | 0.07 | 2.67 | **.008** | | [0.05, 0.34] |
|  | BMI | 0.00 | 0.01 | -0.04 | .966 | | [-0.02, 0.02] |
|  | Income | 0.04 | 0.01 | 5.85 | **< .001** | | [0.03, 0.06] |
| Cigarettes | EWS | 0.88 | 0.30 | 2.97 | **.003** | | [0.30, 1.46] |
|  | Weight Perception | 0.16 | 0.08 | 2.06 | **.040** | | [0.01, 0.31] |
|  | Weight Perception x EWS | -0.21 | 0.09 | -2.39 | **.017** | | [-0.38, -0.04] |
|  | Age | 0.03 | 0.02 | 1.13 | .260 | | [-0.02, 0.07] |
|  | Gender | 0.41 | 0.09 | 4.48 | **< .001** | | [0.23, 0.59] |
|  | BMI | 0.03 | 0.01 | 2.14 | **.033** | | [0.00, 0.05] |
|  | Income | 0.03 | 0.01 | 3.39 | **.001** | | [0.01, 0.05] |
| E-Cig | EWS | 0.37 | 0.25 | 1.49 | .138 | | [-0.12, 0.87] |
|  | Weight Perception | 0.14 | 0.07 | 2.13 | **.034** | | [0.01, 0.27] |
|  | Weight Perception x EWS | -0.08 | 0.07 | -1.04 | .297 | | [-0.22, 0.07] |
|  | Age | 0.02 | 0.02 | 0.85 | .394 | | [-0.02, 0.06] |
|  | Gender | 0.34 | 0.08 | 4.43 | **< .001** | | [0.19, 0.50] |
|  | BMI | -0.00 | 0.01 | -0.20 | .840 | | [-0.02, 0.02] |
|  | Income | 0.04 | 0.01 | 4.99 | **< .001** | | [0.02, 0.05] |
| Breakfast | EWS | -0.46 | 0.38 | -1.20 | .230 | | [-1.20, 0.29] |
|  | Weight Perception | -0.17 | 0.10 | -1.75 | .080 | | [-0.36, 0.02] |
|  | Weight Perception x EWS | 0.06 | 0.11 | 0.53 | .600 | | [-0.16, 0.28] |
|  | Age | -0.05 | 0.03 | -1.59 | .113 | | [-0.11, 0.01] |
|  | Gender | -0.19 | 0.12 | -1.56 | .119 | | [-0.43, 0.05] |
|  | BMI | 0.02 | 0.02 | 1.28 | .201 | | [-0.01, 0.05] |
|  | Income | -0.00 | 0.01 | -0.29 | .776 | | [-0.03, 0.02] |
| Sleep | EWS | 0.69 | 0.85 | 0.82 | .416 | | [-0.97, 2.35] |
|  | Weight Perception | 0.42 | 0.22 | 1.94 | .053 | | [-0.01, 0.85] |
|  | Weight Perception x EWS | 0.05 | 0.25 | 0.20 | .845 | | [-0.44, 0.54] |
|  | Age | 0.04 | 0.07 | 0.59 | .558 | | [-0.10, 0.18] |
|  | Gender | 0.64 | 0.27 | 2.35 | .019 | | [0.11, 1.17] |
|  | BMI | -0.06 | 0.04 | -1.71 | .087 | | [-0.14,0.01] |
|  | Income | -0.05 | 0.03 | -1.76 | .079 | | [-0.10, 0.01] |
| Binge Eating without loss of control | EWS | -1.68 | 0.69 | -2.44 | **.015** | | [-3.04, -0.33] |
|  | Weight Perception | 0.35 | 0.16 | 2.12 | **.034** | | [0.03, 0.67] |
|  | Weight Perception x EWS | 0.66 | 0.20 | 3.32 | **< .001** | | [0.27, 1.04] |
|  | Age | 0.08 | 0.05 | 1.51 | .1315 | | [-0.02, 0.17] |
|  | Gender | 0.07 | 0.19 | 0.34 | .7324 | | [-0.31, 0.44] |
|  | BMI | -0.03 | 0.03 | -1.26 | .2083 | | [-0.09, 0.02] |
|  | Income | -0.00 | 0.02 | -0.20 | .8409 | | [-0.04, 0.03] |
| Binge Eating with loss of control | EWS | -2.03 | 0.85 | -2.38 | **.017** | | [-3.69, -0.36] |
|  | Weight Perception | 0.40 | 0.20 | 2.04 | **.041** | | [0.02, 0.79] |
|  | Weight Perception x EWS | 0.73 | 0.24 | 3.07 | **.002** | | [0.26, 1.19] |
|  | Age | 0.13 | 0.05 | 2.36 | **.018** | | [0.02, 0.23] |
|  | Gender | -0.23 | 0.23 | -1.00 | .317 | | [-0.67, 0.22] |
|  | BMI | -0.03 | 0.03 | -1.08 | .279 | | [-0.09, 0.03] |
|  | Income | 0.01 | 0.02 | 0.52 | .602 | | [-0.03, 0.05] |

Note: Bold indicates statistical significance (*p* < .05). *SE* = standard error; *b* = unstandardised regression coefficient; *β =* standardised regression coefficient and *CI* confidence interval. BMI= Body Mass Index; EWS= Experienced Weight Stigma; UWCB= Unhealthy Weight Control Behaviour; EWUCB= Extremely Unhealthy Weight Control Behaviour; HWCB= Healthy Weight Control Behaviour

Table 13: Moderation of the Association Between Anticipated Weight Stigma and Health Behaviour by Weight Perception

|  |  | | *b* | *SE* | *t* | *p* | 95% CI of *b* |
| --- | --- | --- | --- | --- | --- | --- | --- |
| UWCB | AWS | | -0.01 | 0.02 | -0.73 | .464 | [-0.05, 0.02] |
|  | Weight Perception | | 0.03 | 0.08 | 0.37 | .714 | [-0.13, 0.19] |
|  | Weight Perception x AWS | | 0.01 | 0.00 | 2.75 | **.006** | [0.004, 0.023] |
|  | Age | | 0.07 | 0.02 | 3.08 | **.002** | [0.03, 0.12] |
|  | Gender | | 0.05 | 0.09 | 0.53 | .595 | [-0.13, 0.23] |
|  | BMI | | 0.00 | 0.01 | 0.15 | .883 | [-0.02, 0.03] |
|  | Income | | -0.003 | 0.01 | -0.37 | .708 | [-0.02, 0.01] |
| EUWCB | AWS | | -0.05 | 0.01 | -3.52 | **.001** | [-0.08, -0.02] |
|  | Weight Perception | | -0.23 | 0.06 | -3.59 | **.0004** | [-0.35, -0.10] |
|  | Weight Perception x AWS | | 0.02 | 0.004 | 5.61 | **<.001** | [0.014, 0.029] |
|  | Age | | 0.10 | 0.02 | 5.36 | **<.001** | [0.06, 0.13] |
|  | Gender | | -0.009 | 0.07 | -0.13 | .895 | [-0.15, 0.13] |
|  | BMI | | -0.0007 | 0.009 | -0.07 | .942 | [-0.02, 0.02] |
|  | Income | | -0.005 | 0.007 | -0.74 | .461 | [-0.02, 0.008] |
| HWCB | AWS | | 0.02 | 0.09 | 0.27 | .789 | [-0.16, 0.21] |
|  | Weight Perception | | 0.89 | 0.41 | 2.19 | **.029** | [0.09, 1.69] |
|  | Weight Perception x AWS | | -0.009 | 0.02 | -0.38 | .707 | [-0.06, 0.04] |
|  | Age | | -0.13 | 0.12 | -1.15 | .251 | [-0.36, 0.10] |
|  | Gender | | 0.53 | 0.45 | 1.19 | .236 | [-0.35, 1.41] |
|  | BMI | | 0.16 | 0.06 | 2.68 | .**008** | [0.04, 0.28] |
|  | Income | | 0.08 | 0.04 | 1.91 | .056 | [-0.002, 0.17] |
| Physical Activity | | AWS | 0.76 | 2.57 | 0.29 | .769 | [-4.30, 5.81] |
|  | Weight Perception | | -2.04 | 11.35 | -0.18 | .857 | [-24.33, 20.24] |
|  | Weight Perception x AWS | | -0.84 | 0.68 | -1.23 | .219 | [-2.17, 0.50] |
|  | Age | | -2.32 | 3.26 | -0.71 | .477 | [-8.73, 4.08] |
|  | Gender | | 29.10 | 12.50 | 2.33 | **.020** | [4.57, 53.64] |
|  | BMI | | 4.25 | 1.69 | 2.52 | **.012** | [0.94, 7.56] |
|  | Income | | -2.21 | 1.23 | -1.79 | .073 | [-4.63, 0.21] |
| Alcohol Use | AWS | | 0.021 | 0.016 | 1.30 | .193 | [-0.01, 0.05] |
|  | Weight Perception | | 0.090 | 0.071 | 1.26 | .208 | [-0.05, 0.23] |
|  | Weight Perception x AWS | | -0.0003 | 0.004 | -0.07 | .946 | [-0.009, 0.008] |
|  | Age | | 0.038 | 0.019 | 2.00 | **.046** | [0.001, 0.076] |
|  | Gender | | 0.210 | 0.074 | 2.84 | **.005** | [0.065, 0.355] |
|  | BMI | | -0.004 | 0.010 | -0.39 | .699 | [-0.023, 0.015] |
|  | Income | | 0.042 | 0.007 | 5.81 | **<.001** | [0.028, 0.056] |
| Cigarettes | AWS | | 0.021 | 0.020 | 1.02 | .307 | [-0.019, 0.061] |
|  | Weight Perception | | 0.074 | 0.091 | 0.82 | .414 | [-0.104, 0.252] |
|  | Weight Perception x AWS | | -0.004 | 0.005 | -0.74 | .459 | [-0.015, 0.007] |
|  | Age | | 0.027 | 0.024 | 1.12 | .262 | [-0.020, 0.075] |
|  | Gender | | 0.393 | 0.093 | 4.22 | **<.001** | [0.210, 0.577] |
|  | BMI | | 0.024 | 0.012 | 1.93 | .054 | [-0.0004, 0.048] |
|  | Income | | 0.030 | 0.009 | 3.27 | **.001** | [0.012, 0.048] |
| E-Cig | AWS | | -0.007 | 0.017 | -0.40 | .687 | [-0.041, 0.027] |
|  | Weight Perception | | 0.076 | 0.077 | 0.99 | .323 | [-0.075, 0.226] |
|  | Weight Perception x AWS | | 0.002 | 0.005 | 0.40 | .689 | [-0.007, 0.011] |
|  | Age | | 0.019 | 0.021 | 0.90 | .370 | [-0.022, 0.059] |
|  | Gender | | 0.328 | 0.079 | 4.15 | **<.001** | [0.173, 0.483] |
|  | BMI | | -0.003 | 0.011 | -0.25 | .802 | [-0.023, 0.018] |
|  | Income | | 0.039 | 0.008 | 4.99 | **<.001** | [0.023, 0.054] |
| Breakfast | AWS | | -0.017 | 0.025 | -0.69 | .493 | [-0.067, 0.032] |
|  | Weight Perception | | -0.144 | 0.112 | -1.29 | .199 | [-0.363, 0.076] |
|  | Weight Perception x AWS | | 0.001 | 0.007 | 0.15 | .878 | [-0.012, 0.014] |
|  | Age | | -0.049 | 0.032 | -1.53 | .125 | [-0.112, 0.014] |
|  | Gender | | -0.196 | 0.123 | -1.59 | .112 | [-0.437, 0.046] |
|  | BMI | | 0.023 | 0.017 | 1.39 | .164 | [-0.010, 0.056] |
|  | Income | | -0.001 | 0.012 | -0.10 | .923 | [-0.025, 0.023] |
| Sleep | AWS | | 0.20 | 0.05 | 2.479 | .000 | [0.09, 0.31] |
|  | Weight Perception | | 0.59 | 0.24 | 3.64 | .014 | [0.12, 1.06] |
|  | Weight Perception x AWS | | -0.03 | 0.01 | 2.48 | **.050** | [-0.06, -0.00] |
|  | Age | | 0.03 | 0.07 | -1.97 | .672 | [-0.11, 0.17] |
|  | Gender | | 0.67 | 0.26 | 0.42 | .012 | [0.15,1.19] |
|  | BMI | | -0.08 | 0.04 | 2.53 | .035 | [-0.15, -0.01] |
|  | Income | | -0.05 | 0.03 | -2.12 | .066 | [-0.10, 0.00] |
| Binge Eating without loss of control | AWS | | -0.050 | 0.045 | -1.10 | .272 | [-0.138, 0.039] |
|  | Weight Perception | | 0.306 | 0.192 | 1.60 | .110 | [-0.070, 0.682] |
|  | Weight Perception x AWS | | 0.025 | 0.012 | 2.14 | **.032** | [0.002, 0.048] |
|  | Age | | 0.064 | 0.050 | 1.27 | .205 | [-0.035, 0.162] |
|  | Gender | | 0.114 | 0.194 | 0.59 | .558 | [-0.267, 0.495] |
|  | BMI | | -0.029 | 0.028 | -1.05 | .294 | [-0.083, 0.025] |
|  | Income | | -0.004 | 0.020 | -0.21 | .832 | [-0.043, 0.034] |
| Binge Eating with loss of control | AWS | | -0.041 | 0.053 | -0.78 | .437 | [-0.146, 0.063] |
|  | Weight Perception | | 0.429 | 0.230 | 1.87 | .062 | [-0.022, 0.880] |
|  | Weight Perception x AWS | | 0.022 | 0.014 | 1.61 | .108 | [-0.005, 0.048] |
|  | Age | | 0.116 | 0.054 | 2.16 | **.031** | [0.011, 0.221] |
|  | Gender | | -0.190 | 0.226 | -0.84 | .400 | [-0.632, 0.252] |
|  | BMI | | -0.023 | 0.029 | -0.79 | .427 | [-0.081, 0.034] |
|  | Income | | 0.015 | 0.022 | 0.66 | .510 | [-0.029, 0.058] |

Note: Bold indicates statistical significance (*p* < .05). *SE* = standard error; *b* = unstandardised regression coefficient; *β =* standardised regression coefficient and *CI* confidence interval. BMI= Body Mass Index; AWS= Anticipated Weight Stigma; UWCB= Unhealthy Weight Control Behaviour; EWUCB= Extremely Unhealthy Weight Control Behaviour; HWCB= Healthy Weight Control Behaviour

Table14: Moderation of the Association Between Internalised Weight stigma and Health Behaviour by Weight Perception

|  |  | | *b* | *SE* | *t* (or Z) | *p* | 95% CI of *b* |
| --- | --- | --- | --- | --- | --- | --- | --- |
| UWCB | IWS | | -0.01 | 0.01 | -0.81 | .419 | [-0.02, 0.01] |
|  | Weight Perception | | -0.01 | 0.09 | -0.09 | .927 | [-0.18, 0.16] |
|  | Weight Perception x IWS | | **0.01** | **0.00** | **2.67** | **.008** | **[0.00, 0.01]** |
|  | Age | | **0.07** | **0.02** | **2.97** | **.003** | **[0.02, 0.12]** |
|  | Gender | | 0.01 | 0.09 | 0.16 | .870 | [-0.16, 0.19] |
|  | BMI | | 0.01 | 0.01 | 0.54 | .590 | [-0.02, 0.03] |
|  | Income | | -0.00 | 0.01 | -0.23 | .819 | [-0.02, 0.02] |
| EUWCB | IWS | | **-0.02** | **0.01** | **-3.04** | **.003** | **[-0.03, -0.01]** |
|  | Weight Perception | | **-0.24** | **0.07** | **-3.63** | **<.001** | **[-0.36, -0.11]** |
|  | Weight Perception x IWS | | **0.01** | **0.00** | **5.14** | **<.001** | **[0.00, 0.01]** |
|  | Age | | **0.10** | **0.02** | **5.40** | **<.001** | **[0.06, 0.13]** |
|  | Gender | | -0.02 | 0.07 | -0.28 | .777 | [-0.15, 0.12] |
|  | BMI | | 0.00 | 0.01 | 0.35 | .729 | [-0.02, 0.02] |
|  | Income | | -0.01 | 0.01 | -0.85 | .398 | [-0.02, 0.01] |
| HWCB | IWS | | 0.04 | 0.04 | 1.17 | .241 | [-0.03, 0.12] |
|  | Weight Perception | | **1.05** | **0.42** | **2.51** | **.013** | **[0.23, 1.87]** |
|  | Weight Perception x IWS | | -0.01 | 0.01 | -1.09 | .278 | [-0.03, 0.01] |
|  | Age | | -0.15 | 0.12 | -1.29 | .196 | [-0.38, 0.08] |
|  | Gender | | 0.54 | 0.44 | 1.21 | .226 | [-0.33, 1.41] |
|  | BMI | | **0.16** | **0.06** | **2.75** | **.006** | **[0.05, 0.28]** |
|  | Income | | 0.08 | 0.04 | 1.84 | .066 | [-0.01, 0.17] |
| Physical Activity | | IWS | 1.93 | 1.05 | 1.84 | .066 | [-0.12, 3.98] |
|  | Weight Perception | | 6.01 | 11.80 | 0.51 | .611 | [-17.15, 29.17] |
|  | Weight Perception x IWS | | **-0.65** | **0.28** | **-2.34** | **.020** | **[-1.19, -0.10]** |
|  | Age | | -2.87 | 3.27 | -0.88 | .381 | [-9.29, 3.56] |
|  | Gender | | **31.76** | **12.47** | **2.55** | **.011** | **[7.28, 56.24]** |
|  | BMI | | **3.99** | **1.68** | **2.38** | **.018** | **[0.69, 7.28]** |
|  | Income | | **-2.47** | **1.23** | **-2.02** | **.044** | **[-4.88, -0.07]** |
| Alcohol Use | IWS | | 0.00 | 0.01 | 0.45 | .650 | [-0.01, 0.02] |
|  | Weight Perception | | 0.08 | 0.08 | 1.05 | .296 | [-0.07, 0.23] |
|  | Weight Perception x IWS | | 0.00 | 0.00 | 0.30 | .768 | [-0.00, 0.00] |
|  | Age | | **0.04** | **0.02** | **2.04** | **.042** | **[0.00, 0.08]** |
|  | Gender | | **0.19** | **0.07** | **2.57** | **.010** | **[0.05, 0.34]** |
|  | BMI | | -0.00 | 0.01 | -0.13 | .895 | [-0.02, 0.02] |
|  | Income | | **0.04** | **0.01** | **5.80** | **<.001** | **[0.03, 0.06]** |
| Cigarettes | IWS | | 0.01 | 0.01 | 0.61 | .541 | [-0.01, 0.02] |
|  | Weight Perception | | 0.06 | 0.10 | 0.65 | .518 | [-0.13, 0.25] |
|  | Weight Perception x IWS | | -0.00 | 0.00 | -0.46 | .645 | [-0.01, 0.00] |
|  | Age | | 0.03 | 0.02 | 1.16 | .245 | [-0.02, 0.08] |
|  | Gender | | **0.39** | **0.09** | **4.18** | **<.001** | **[0.21, 0.57]** |
|  | BMI | | **0.02** | **0.01** | **1.96** | **.050** | **[0.00, 0.05]** |
|  | Income | | **0.03** | **0.01** | **3.30** | **.001** | **[0.01, 0.05]** |
| E-Cigarettes | IWS | | 0.00 | 0.01 | 0.17 | .865 | [-0.01, 0.02] |
|  | Weight Perception | | 0.08 | 0.08 | 1.02 | .308 | [-0.08, 0.24] |
|  | Weight Perception x IWS | | 0.00 | 0.00 | 0.05 | .961 | [-0.00, 0.00] |
|  | Age | | 0.02 | 0.02 | 0.87 | .382 | [-0.02, 0.06] |
|  | Gender | | **0.34** | **0.08** | **4.29** | **<.001** | **[0.18, 0.49]** |
|  | BMI | | -0.00 | 0.01 | -0.30 | .767 | [-0.02, 0.02] |
|  | Income | | **0.04** | **0.01** | **4.95** | **<.001** | **[0.02, 0.05]** |
| Breakfast | IWS | | -0.00 | 0.01 | -0.09 | .931 | [-0.02, 0.02] |
|  | Weight Perception | | -0.08 | 0.12 | -0.73 | .465 | [-0.31, 0.14] |
|  | Weight Perception x IWS | | -0.00 | 0.00 | -0.37 | .710 | [-0.01, 0.00] |
|  | Age | | -0.05 | 0.03 | -1.55 | .122 | [-0.11, 0.01] |
|  | Gender | | -0.18 | 0.12 | -1.46 | .145 | [-0.42, 0.06] |
|  | BMI | | 0.02 | 0.02 | 1.35 | .178 | [-0.01, 0.05] |
|  | Income | | -0.00 | 0.01 | -0.25 | .803 | [-0.03, 0.02] |
| Sleep | IWS | | 0.09 | 0.02 | 4.28 | **<.001** | [0.051, 0.13] |
|  | Weight Perception | | 0.54 | 0.25 | 2.26 | **.024** | [0.07, 1.05] |
|  | Weight Perception x IWS | | -0.01 | 0.01 | -2.27 | **.023** | [-0.02,-0.00] |
|  | Age | | 0.03 | 0.07 | 0.39 | .691 | [-0.10,0.16] |
|  | Gender | | 0.66 | 0.26 | 2.50 | **.013** | [0.14, 1.17] |
|  | BMI | | -0.07 | 0.03 | -1.97 | **.049** | [-0.14, -0.00] |
|  | Income | | -0.05 | 0.03 | -2.05 | **.041** | [-0.10,-0.00] |
| Binge Eating without loss of control | IWS | | -0.00 | 0.02 | -0.27 | .790 | [-0.04, 0.03] |
|  | Weight Perception | | 0.39 | 0.20 | 1.93 | .053 | [-0.01, 0.79] |
|  | Weight Perception x IWS | | 0.01 | 0.00 | 1.27 | .202 | [-0.00, 0.02] |
|  | Age | | 0.07 | 0.05 | 1.35 | .178 | [-0.03, 0.17] |
|  | Gender | | 0.08 | 0.19 | 0.43 | .667 | [-0.29, 0.46] |
|  | BMI | | -0.02 | 0.03 | -0.86 | .390 | [-0.08, 0.03] |
|  | Income | | -0.00 | 0.02 | -0.20 | .839 | [-0.04, 0.03] |
| Binge Eating with loss of control | IWS | | -0.01 | 0.02 | -0.33 | .738 | [-0.05, 0.04] |
|  | Weight Perception | | 0.46 | 0.24 | 1.90 | .058 | [-0.02, 0.94] |
|  | Weight Perception x IWS | | 0.01 | 0.01 | 1.17 | .241 | [-0.00, 0.02] |
|  | Age | | 0.12 | 0.05 | 2.20 | **.028** | [0.01, 0.22] |
|  | Gender | | -0.21 | 0.23 | -0.92 | .357 | [-0.66, 0.24] |
|  | BMI | | -0.02 | 0.03 | -0.65 | .515 | [-0.08, 0.04] |
|  | Income | | 0.01 | 0.02 | 0.55 | .581 | [-0.03, 0.06] |

Note: Bold indicates statistical significance (*p* < .05). *SE* = standard error; *b* = unstandardised regression coefficient; *β =* standardised regression coefficient; and *CI* confidence interval. BMI= Body Mass Index; IWS= Internalised Weight Stigma; UWCB= Unhealthy Weight Control Behaviour; EWUCB= Extremely Unhealthy Weight Control Behaviour; HWCB= Healthy Weight Control Behaviour

Table 15: Moderation of the Association Between Experienced Weight Stigma and Health Behaviour by BMI

|  |  | B (Log-Odds) | SE | t (Z) | p | 95% CI of b |
| --- | --- | --- | --- | --- | --- | --- |
| UWCB | EWS | -0.35 | 0.43 | -0.83 | .409 | [-1.19, 0.48] |
|  | BMI | 0.03 | 0.02 | 1.92 | **.055** | [-0.001, 0.06] |
|  | BMI **x** EWS | 0.03 | 0.02 | 1.67 | .095 | [-0.01, 0.07] |
|  | Age | 0.07 | 0.02 | 2.71 | **.007** | [0.02, 0.11] |
|  | Gender | -0.07 | 0.09 | -0.80 | .424 | [-0.26, 0.11] |
|  | Income | 0.00 | 0.01 | -0.00 | .996 | [-0.02, 0.02] |
| EUWCB | EWS | -0.59 | 0.32 | -1.85 | .065 | [-1.22, 0.04] |
|  | BMI | -0.00 | 0.01 | -0.36 | .716 | [-0.03, 0.02] |
|  | BMI x EWS | 0.04 | 0.01 | 2.84 | **.005** | [0.01, 0.07] |
|  | Age | 0.10 | 0.02 | 5.21 | **.000** | [0.06, 0.13] |
|  | Gender | -0.06 | 0.07 | -0.87 | .383 | [-0.20, 0.08] |
|  | Income | -0.00 | 0.01 | -0.71 | .479 | [-0.02, 0.01] |
| HWCB | EWS | 2.32 | 2.02 | 1.15 | .252 | [-1.65, 6.29] |
|  | BMI | 0.37 | 0.07 | 5.00 | **.000** | [0.23, 0.52] |
|  | BMI x EWS | -0.12 | 0.09 | -1.40 | .162 | [-0.30, 0.05] |
|  | Age | -0.17 | 0.12 | -1.49 | .136 | [-0.40, 0.05] |
|  | Gender | 0.24 | 0.44 | 0.56 | .577 | [-0.62, 1.11] |
|  | Income | 0.09 | 0.04 | 1.96 | .051 | [-0.00, 0.17] |
| Physical Activity | EWS | 116.50 | 72.97 | 1.60 | .111 | [-26.76, 259.77] |
|  | BMI | 9.69 | 56.73 | 0.17 | .865 | [-101.70, 121.07] |
|  | BMI x EWS | 2.70 | 2.09 | 1.29 | .196 | [-1.40, 6.80] |
|  | Age | -1.96 | 2.49 | -0.78 | .433 | [-6.85, 2.94] |
|  | Gender | -2.46 | 3.27 | -0.75 | .451 | [-8.88, 3.95] |
|  | Income | 35.67 | 12.30 | 2.90 | **.004** | [11.52, 59.82] |
| Alcohol Use | EWS | 0.05 | 0.34 | 0.16 | .873 | [-0.61, 0.72] |
|  | BMI | 0.01 | 0.01 | 1.04 | .300 | [-0.01, 0.04] |
|  | BMI x EWS | 0.01 | 0.01 | 0.48 | .631 | [-0.02, 0.04] |
|  | Age | 0.04 | 0.02 | 2.01 | .045 | [0.001, 0.08] |
|  | Gender | 0.16 | 0.07 | 2.22 | **.027** | [0.02, 0.31] |
|  | Income | 0.04 | 0.01 | 5.89 | **<.001** | [0.03, 0.06] |
| Cigarettes | EWS | 0.80 | 0.42 | 1.91 | .056 | [-0.02, 1.62] |
|  | BMI | 0.04 | 0.02 | 2.88 | **.004** | [0.01, 0.08] |
|  | BMI x EWS | -0.03 | 0.02 | -1.46 | .144 | [-0.06, 0.01] |
|  | Age | 0.03 | 0.02 | 1.18 | .240 | [-0.02, 0.08] |
|  | Gender | 0.39 | 0.09 | 4.35 | **<.001** | [0.22, 0.57] |
|  | Income | 0.03 | 0.01 | 3.37 | **.001** | [0.01, 0.05] |
| E-Cig | EWS | 0.45 | 0.36 | 1.27 | .204 | [-0.25, 1.15] |
|  | BMI | 0.02 | 0.01 | 1.52 | .128 | [-0.01, 0.05] |
|  | BMI x EWS | -0.01 | 0.02 | -0.93 | .355 | [-0.05, 0.02] |
|  | Age | 0.02 | 0.02 | 0.78 | .433 | [-0.02, 0.06] |
|  | Gender | 0.31 | 0.08 | 4.09 | <.001 | [0.16, 0.47] |
|  | Income | 0.04 | 0.01 | 5.04 | <.001 | [0.02, 0.05] |
| Breakfast | EWS | 0.06 | 0.56 | 0.11 | .916 | [-1.03, 1.15] |
|  | BMI | 0.01 | 0.02 | 0.59 | .558 | [-0.03, 0.05] |
|  | BMI x EWS | -0.02 | 0.02 | -0.62 | .539 | [-0.06, 0.03] |
|  | Age | -0.05 | 0.03 | -1.51 | .131 | [-0.11, 0.01] |
|  | Gender | -0.15 | 0.12 | -1.28 | .203 | [-0.39, 0.08] |
|  | Income | -0.00 | 0.01 | -0.32 | .749 | [-0.03, 0.02] |
| Sleep | EWS | -0.71 | 1.24 | -0.58 | .565 | [-3.15,1.72] |
|  | BMI | -0.04 | 0.05 | -0.92 | .358 | [-0.13, 0.05] |
|  | BMI x EWS | 0.07 | 0.06 | 1.33 | .183 | [-0.03, 0.18] |
|  | Age | 0.04 | 0.07 | 0.55 | .583 | [-0.10, 0.18] |
|  | Gender | 0.53 | 0.27 | 1.98 | .048 | [0.00, 1.06] |
|  | Income | -0.05 | 0.03 | -1.68 | .094 | [-0.10, 0.01] |
| Binge Eating without loss of control | EWS | -1.18 | 0.89 | -1.32 | .187 | [-2.93, 0.57] |
|  | BMI | 0.04 | 0.03 | 1.12 | .264 | [-0.03, 0.10] |
|  | BMI x EWS | 0.08 | 0.04 | 2.01 | **.045** | [0.002, 0.15] |
|  | Age | 0.05 | 0.05 | 1.08 | .281 | [-0.04, 0.15] |
|  | Gender | -0.12 | 0.19 | -0.65 | .518 | [-0.49, 0.25] |
|  | Income | -0.0003 | 0.02 | -0.02 | .987 | [-0.04, 0.04] |
| Binge Eating with loss of control | EWS | -1.39 | 0.99 | -1.41 | .159 | [-3.33, 0.55] |
|  | BMI | 0.04 | 0.03 | 1.09 | .275 | [-0.03, 0.11] |
|  | BMI xEWS | 0.09 | 0.04 | 2.08 | **.038** | [0.01, 0.17] |
|  | Age | 0.11 | 0.05 | 2.00 | **.045** | [0.002, 0.21] |
|  | Gender | -0.45 | 0.23 | -1.99 | **.046** | [-0.90, -0.01] |
|  | Income | 0.01 | 0.02 | 0.70 | .482 | [-0.03, 0.06] |

Note: Bold indicates statistical significance (*p* < .05). *SE* = standard error; *b* = unstandardised regression coefficient; *β =* standardised regression coefficient; and *CI* confidence interval. BMI= Body Mass Index; EWS= Experienced Weight Stigma; UWCB= Unhealthy Weight Control Behaviour; EWUCB= Extremely Unhealthy Weight Control Behaviour; HWCB= Healthy Weight Control Behaviour

Table 16 : Moderation of the Association Between Anticipated Weight Stigma and Health Behaviour by BMI

|  |  | B (Log-Odds) | | SE | t /Z | p | 95% CI of b |
| --- | --- | --- | --- | --- | --- | --- | --- |
| UWCB | AWS | | 0.01 | 0.02 | 0.20 | .840 | [-0.04, 0.05] |
|  | BMI | | 0.01 | 0.02 | 0.69 | .488 | [-0.02, 0.05] |
|  | BMI **x** AWS | | 0.00 | 0.00 | 1.42 | .156 | [-0.001, 0.004] |
|  | Age | | 0.07 | 0.02 | 2.76 | **.006** | [0.02, 0.11] |
|  | Gender | | 0.01 | 0.09 | 0.06 | .956 | [-0.17, 0.18] |
|  | Income | | -0.00 | 0.01 | -0.29 | .769 | [-0.02, 0.02] |
| EUWCB | AWS | | -0.05 | 0.02 | -2.76 | **.006** | [-0.09, -0.02] |
|  | BMI | | -0.04 | 0.01 | -2.82 | **.005** | [-0.06, -0.01] |
|  | BMI x AWS | | 0.003 | 0.001 | 4.29 | **<.001** | [0.002, 0.005] |
|  | Age | | 0.09 | 0.02 | 5.15 | **<.001** | [0.06, 0.13] |
|  | Gender | | 0.00 | 0.07 | -0.01 | .995 | [-0.14, 0.14] |
|  | Income | | -0.005 | 0.007 | -0.69 | .493 | [-0.02, 0.01] |
| HWCB | AWS | | 0.20 | 0.12 | 1.63 | .104 | [-0.04, 0.44] |
|  | BMI | | 0.39 | 0.09 | 4.44 | **<.001** | [0.22, 0.56] |
|  | BMI x AWS | | -0.01 | 0.01 | -1.60 | .109 | [-0.02, 0.002] |
|  | Age | | -0.15 | 0.12 | -1.32 | .189 | [-0.38, 0.08] |
|  | Gender | | 0.28 | 0.45 | 0.62 | .534 | [-0.60, 1.15] |
|  | Income | | 0.09 | 0.04 | 1.93 | .055 | [-0.002, 0.17] |
| Physical Activity | AWS | | 3.39 | 3.38 | 1.00 | .316 | [-3.24, 10.03] |
|  | BMI | | 5.93 | 2.42 | 2.45 | **.015** | [1.18, 10.68] |
|  | BMI x AWS | | -0.25 | 0.14 | -1.79 | .074 | [-0.52, 0.02] |
|  | Age | | -1.85 | 3.25 | -0.57 | .570 | [-8.24, 4.54] |
|  | Gender | | 30.41 | 12.42 | 2.45 | **.015** | [6.01, 54.80] |
|  | Income | | -2.31 | 1.23 | -1.88 | .061 | [-4.73, 0.11] |
| Alcohol Use | AWS | | 0.032 | 0.020 | 1.57 | .116 | [-0.008, 0.072] |
|  | BMI | | 0.014 | 0.015 | 0.97 | .333 | [-0.015, 0.043] |
|  | BMI x AWS | | -0.0004 | 0.0008 | -0.50 | .615 | [-0.0021, 0.0012] |
|  | Age | | 0.037 | 0.019 | 1.91 | .056 | [-0.001, 0.074] |
|  | Gender | | 0.186 | 0.074 | 2.52 | **.012** | [0.041, 0.331] |
|  | Income | | 0.042 | 0.007 | 5.83 | **<.001** | [0.028, 0.057] |
| Cigarettes | AWS | | 0.02 | 0.03 | 0.66 | .509 | [-0.03, 0.07] |
|  | BMI | | 0.03 | 0.02 | 1.75 | .080 | [-0.00, 0.07] |
|  | BMI x AWS | | -0.00 | 0.00 | -0.40 | .688 | [-0.00, 0.00] |
|  | Age | | 0.03 | 0.02 | 1.14 | .255 | [-0.02, 0.08] |
|  | Gender | | 0.39 | 0.09 | 4.18 | **<.001** | [0.21, 0.57] |
|  | Income | | 0.02 | 0.03 | 0.66 | .509 | [-0.03, 0.07] |
| E-Cig | IWS | | 0.02 | 0.02 | 1.09 | .274 | [-0.02, 0.07] |
|  | BMI | | 0.03 | 0.02 | 1.61 | .108 | [-0.01, 0.06] |
|  | BMI x AWS | | -0.00 | 0.00 | -1.02 | .307 | [-0.00, 0.00] |
|  | Age | | 0.02 | 0.02 | 0.77 | .441 | [-0.02, 0.06] |
|  | Gender | | 0.29 | 0.08 | 3.73 | **<.001** | [0.14, 0.45] |
|  | Income | | 0.04 | 0.01 | 5.00 | **<.001** | [0.02, 0.05] |
| Breakfast | AWS | | 0.01 | 0.03 | 0.29 | .770 | [-0.06, 0.07] |
|  | BMI | | 0.02 | 0.02 | 0.90 | .368 | [-0.03, 0.07] |
|  | BMI x AWS | | -0.00 | 0.00 | -0.80 | .423 | [-0.00, 0.00] |
|  | Age | | -0.05 | 0.03 | -1.43 | .152 | [-0.11, 0.02] |
|  | Gender | | -0.17 | 0.12 | -1.43 | .153 | [-0.41, 0.07] |
|  | Income | | -0.00 | 0.01 | -0.18 | .854 | [-0.03, 0.02] |
| Sleep | AWS | | 0.1 | 0.1 | 1.7 | 0.096 | [-0.02, 0.26] |
|  | BMI | | 0.0 | 0.1 | -0.8 | 0.438 | [-0.14, 0.06 |
|  | BMI x AWS | | 0.0 | 0.0 | -0.2 | 0.805 | [-0.01, 0.06 |
|  | Age | | 0.0 | 0.1 | 0.5 | 0.649 | [-0.10, 0.01] |
|  | Gender | | 0.6 | 0.3 | 2.4 | 0.017 | [0.11, 1.15] |
|  | Income | | 0.0 | 0.0 | -1.7 | 0.083 | [-0.10, 0.01] |
| Binge Eating without loss of control | AWS | | 0.02 | 0.06 | 0.36 | .715 | [-0.09, 0.13] |
|  | BMI | | 0.04 | 0.04 | 1.04 | .299 | [-0.04, 0.12] |
|  | BMI x AWS | | 0.00 | 0.00 | 0.65 | .514 | [-0.00, 0.01] |
|  | Age | | 0.04 | 0.05 | 0.86 | .391 | [-0.05, 0.14] |
|  | Gender | | -0.03 | 0.19 | -0.14 | .890 | [-0.40, 0.35] |
|  | Income | | -0.00 | 0.02 | -0.12 | .907 | [-0.04, 0.04] |
| Binge Eating with loss of control | AWS | | 0.04 | 0.06 | 0.72 | .473 | [-0.08, 0.16] |
|  | BMI | | 0.06 | 0.04 | 1.38 | .169 | [-0.03, 0.14] |
|  | BMI x AWS | | 0.00 | 0.00 | 0.28 | .781 | [-0.00, 0.01] |
|  | Age | | 0.10 | 0.05 | 1.85 | .065 | [-0.01, 0.20] |
|  | Gender | | -0.34 | 0.23 | -1.50 | .133 | [-0.79, 0.10] |
|  | Income | | 0.02 | 0.02 | 0.74 | .462 | [-0.03, 0.06] |

Note: Bold indicates statistical significance (*p* < .05). *SE* = standard error; *b* = unstandardised regression coefficient; *β =* standardised regression coefficient; and *CI* confidence interval. BMI= Body Mass Index; AWS= Anticipated Weight Stigma; UWCB= Unhealthy Weight Control Behaviour; EWUCB= Extremely Unhealthy Weight Control Behaviour; HWCB= Healthy Weight Control Behaviour

Table 17 : Moderation of the Association Between Internalised Weight Stigma and Health Behaviour by BMI

|  |  | b (Log-Odds) | | SE | t (Z) | p | 95% CI of b |
| --- | --- | --- | --- | --- | --- | --- | --- |
| UWCB | IWS | | -0.01 | 0.01 | -1.01 | .310 | [-0.03, 0.01] |
|  | BMI | | -0.01 | 0.02 | -0.33 | .740 | [-0.04, 0.03] |
|  | BMI **x** IWS | | 0.001 | 0.000 | 2.55 | **.010** | [0.000, 0.002] |
|  | Age | | 0.07 | 0.02 | 2.73 | **.006** | [0.02, 0.11] |
|  | Gender | | -0.02 | 0.09 | -0.21 | .830 | [-0.20, 0.16] |
|  | Income | | -0.001 | 0.01 | -0.16 | .874 | [-0.02, 0.02] |
| EUWCB | IWS | | -0.03 | 0.01 | -3.45 | **.001** | [-0.04, -0.01] |
|  | BMI | | -0.05 | 0.01 | -3.46 | **.001** | [-0.07, -0.02] |
|  | BMI x IWS | | 0.002 | 0.000 | 5.00 | **<.001** | [0.001, 0.002] |
|  | Age | | 0.10 | 0.02 | 5.32 | **<.001** | [0.06, 0.13] |
|  | Gender | | -0.01 | 0.07 | -0.22 | .83 | [-0.15, 0.12] |
|  | Income | | 0.00 | 0.01 | -0.80 | .43 | [-0.02, 0.01] |
| HWCB | IWS | | 0.12 | 0.05 | 2.42 | **.016** | [0.02, 0.22] |
|  | BMI | | 0.41 | 0.09 | 4.83 | **<.001** | [0.25, 0.58] |
|  | BMI x IWS | | -0.0045 | 0.002 | -2.22 | **.027** | [-0.009, -0.001] |
|  | Age | | -0.17 | 0.12 | -1.46 | .144 | [-0.40, 0.06] |
|  | Gender | | 0.31 | 0.44 | 0.70 | .484 | [-0.56, 1.17] |
|  | Income | | 0.08 | 0.04 | 1.84 | .066 | [-0.01, 0.17] |
| Physical Activity | IWS | | 2.50 | 1.39 | 1.81 | .071 | [-0.22, 5.22] |
|  | BMI | | 6.20 | 2.41 | 2.57 | **.010** | [1.47, 10.94] |
|  | BMI x IWS | | -0.13 | 0.06 | -2.26 | **.024** | [-0.24, -0.02] |
|  | Age | | -2.35 | 3.27 | -0.72 | .472 | [-8.77, 4.07] |
|  | Gender | | 34.64 | 12.38 | 2.80 | **.005** | [10.33, 58.96] |
|  | Income | | -2.53 | 1.23 | -2.06 | **.039** | [-4.95, -0.12] |
| Alcohol Use | IWS | | -0.003 | 0.008 | -0.35 | .729 | [-0.02, 0.01] |
|  | BMI | | -0.002 | 0.015 | -0.15 | .884 | [-0.03, 0.03] |
|  | BMI x IWS | | 0.0004 | 0.0003 | 1.11 | .268 | [-0.0003, 0.0011] |
|  | Age | | 0.038 | 0.019 | 1.98 | **.048** | [0.0004, 0.076] |
|  | Gender | | 0.175 | 0.074 | 2.38 | **.018** | [0.031, 0.32] |
|  | Income | | 0.043 | 0.007 | 5.86 | **<.001** | [0.028, 0.057] |
| Cigarettes | IWS | | -0.007 | 0.01 | -0.69 | .489 | [-0.03, 0.01] |
|  | BMI | | 0.013 | 0.02 | 0.73 | .467 | [-0.02, 0.05] |
|  | BMI x IWS | | 0.0004 | 0.0004 | 0.88 | .378 | [-0.0005, 0.0012] |
|  | Age | | 0.029 | 0.02 | 1.21 | .226 | [-0.02, 0.08] |
|  | Gender | | 0.394 | 0.09 | 4.30 | **<.001** | [0.21, 0.57] |
|  | Income | | 0.030 | 0.01 | 3.33 | **.001** | [0.01, 0.05] |
| E-Cig | IWS | | 0.005 | 0.009 | 0.51 | .610 | [-0.01, 0.02] |
|  | BMI | | 0.011 | 0.02 | 0.72 | .473 | [-0.02, 0.04] |
|  | BMI x IWS | | -0.0001 | 0.0004 | -0.22 | .826 | [-0.0008, 0.0006] |
|  | Age | | 0.016 | 0.02 | 0.79 | .428 | [-0.02, 0.06] |
|  | Gender | | 0.315 | 0.08 | 4.05 | **<.001** | [0.16, 0.47] |
|  | Income | | 0.038 | 0.01 | 4.98 | **<.001** | [0.02, 0.05] |
| Breakfast | IWS | | 0.002 | 0.01 | 0.16 | .876 | [-0.02, 0.03] |
|  | BMI | | 0.018 | 0.02 | 0.75 | .456 | [-0.03, 0.06] |
|  | BMI x IWS | | -0.0003 | 0.0006 | -0.59 | .553 | [-0.00, 0.00] |
|  | Age | | -0.047 | 0.03 | -1.46 | .146 | [-0.11, 0.02] |
|  | Gender | | -0.154 | 0.12 | -1.27 | .204 | [-0.39, 0.08] |
|  | Income | | -0.003 | 0.01 | -0.28 | .777 | [-0.03, 0.02] |
| Sleep | IWS | | 0.06 | 0.03 | 2.05 | .041 | [0.00, 0.12] |
|  | BMI | | -0.04 | 0.05 | -0.83 | .406 | [-0.14, 0.06] |
|  | BMI x IWS | | 0.00 | 0.00 | -0.40 | .691 | [0.00,0.00] |
|  | Age | | 0.03 | 0.07 | 0.49 | .628 | [-0.10, 0.17] |
|  | Gender | | 0.66 | 0.26 | 2.53 | .012 | [0.15, 1.18] |
|  | Income | | -0.05 | 0.03 | -1.99 | .047 | [-0.10,0.00] |
| Binge Eating without loss of control | IWS | | -0.002 | 0.023 | -0.08 | .936 | [-0.05, 0.04] |
|  | BMI | | 0.019 | 0.038 | 0.51 | .609 | [-0.06, 0.09] |
|  | BMI x IWS | | 0.0011 | 0.0009 | 1.15 | .248 | [-0.0008, 0.003] |
|  | Age | | 0.05 | 0.049 | 1.02 | .309 | [-0.05, 0.15] |
|  | Gender | | -0.05 | 0.19 | -0.25 | .803 | [-0.42, 0.32] |
|  | Income | | -0.003 | 0.019 | -0.15 | .883 | [-0.04, 0.03] |
| Binge Eating with loss of control | IWS | | 0.00 | 0.02 | 0.20 | .845 | [-0.04,0.52] |
|  | BMI | | 0.03 | 0.04 | 0.77 | .442 | [-0.05,0.11] |
|  | BMI x IWS | | 0.00 | 0.00 | 0.91 | .365 | [-0.00,0.00] |
|  | Age | | 0.10 | 0.05 | 1.96 | .050 | [-0.00,0.21] |
|  | Gender | | -0.36 | 0.23 | -1.56 | .119 | [-0.81,0.09 |
|  | Income | | 0.01 | 0.02 | 0.58 | .561 | [-0.03,0.05] |

Note: Bold indicates statistical significance (*p* < .05). *SE* = standard error; *b* = unstandardised regression coefficient; *β =* standardised regression coefficient; and *CI* confidence interval. BMI= Body Mass Index; IWS= Internalised Weight Stigma; UWCB= Unhealthy Weight Control Behaviour; EWUCB= Extremely Unhealthy Weight Control Behaviour; HWCB= Healthy Weight Control Behaviour
